# The Ever-Evolving Concept of the Gene: The Use of RNA/Protein Experimental Techniques to Understand Genome Functions

**DOI:** 10.3389/fmolb.2018.00020

**Published:** 2018-03-06

**Authors:** Andrea Cipriano, Monica Ballarino

**Affiliations:** Department of Biology and Biotechnology Charles Darwin, Sapienza University of Rome, Rome, Italy

**Keywords:** gene, genomics, transcriptomics, long noncoding RNA, RNA-protein interactions, RNA-seq, RNA pull-down, CLIP

## Abstract

The completion of the human genome sequence together with advances in sequencing technologies have shifted the paradigm of the genome, as composed of discrete and hereditable coding entities, and have shown the abundance of functional noncoding DNA. This part of the genome, previously dismissed as “junk” DNA, increases proportionally with organismal complexity and contributes to gene regulation beyond the boundaries of known protein-coding genes. Different classes of functionally relevant nonprotein-coding RNAs are transcribed from noncoding DNA sequences. Among them are the long noncoding RNAs (lncRNAs), which are thought to participate in the basal regulation of protein-coding genes at both transcriptional and post-transcriptional levels. Although knowledge of this field is still limited, the ability of lncRNAs to localize in different cellular compartments, to fold into specific secondary structures and to interact with different molecules (RNA or proteins) endows them with multiple regulatory mechanisms. It is becoming evident that lncRNAs may play a crucial role in most biological processes such as the control of development, differentiation and cell growth. This review places the evolution of the concept of the gene in its historical context, from Darwin's hypothetical mechanism of heredity to the post-genomic era. We discuss how the original idea of protein-coding genes as unique determinants of phenotypic traits has been reconsidered in light of the existence of noncoding RNAs. We summarize the technological developments which have been made in the genome-wide identification and study of lncRNAs and emphasize the methodologies that have aided our understanding of the complexity of lncRNA-protein interactions in recent years.

## From gene to genome, an evolution in the thinking

### The meaning of “gene”

**Figure 1:**
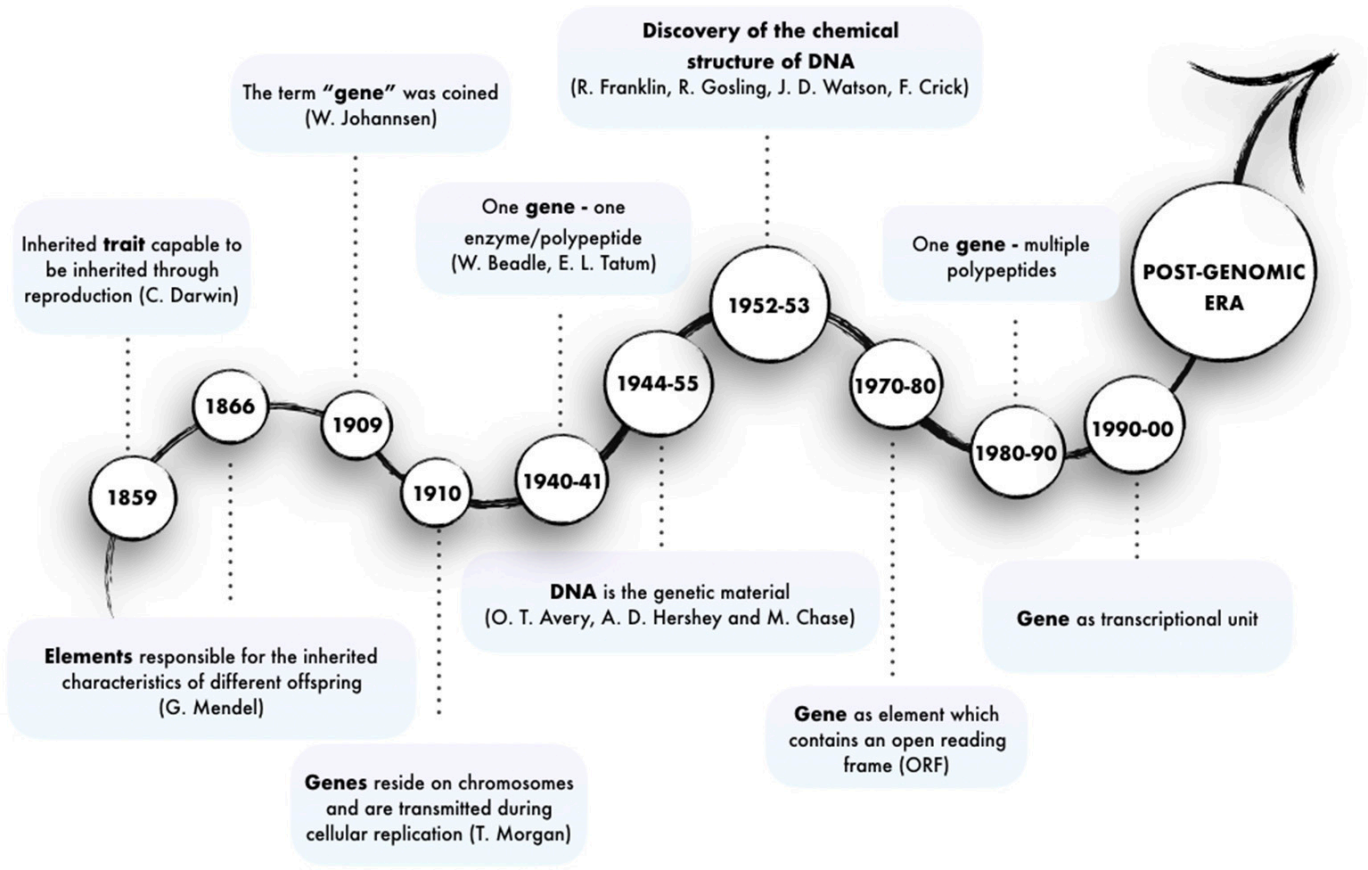
Major steps in the evolution of the concept of gene.

At first sight, the question “what is a gene?” would seem to elicit a simple answer: genes transmit inherited characteristics and must be the cause of them. However, since the time when the idea of gene was first mooted, the advent of novel Next-Generation Sequencing (NGS) technologies have complicated and expanded this view into an ever-evolving concept (Figure 1).

The existence of genetic traits inherited as discrete entities was first intuited by (Darwin, 1859) and (Mendel, 1866) in the mid-nineteenth century. However, the term “gene” was first used by the plant physiologist and geneticist von Wilhelm Johannsen in his book “Elemente der exakten Erblichkeitslehre.” He refers to “*conditions, foundations and determiners which are present in unique, separate and thereby independent ways by which many characteristics of the organisms are specified (…) precisely what we wish to call genes*” (Johannsen, 1909), thus also ascribing to genes the responsibility for phenotypes. The term was inspired by “pangene,” which was used by Hugo de Vries for entities involved in Pangenesis (Heimans, 1962). This abstract concept was given concrete substance in 1910 by Thomas Hunt Morgan, who showed that genes are subcellular particles residing on specific structures, the chromosomes, which are transmitted through cellular replication (Morgan et al., 1915). Later in 1941, George Wells Beadle and Edward Lawrie Tatum demonstrated that mutations in genes caused errors in specific steps of metabolic pathways. This made it possible for the first time to link the concept of gene to the synthesis of enzymes, yielding the “one gene–one enzyme” paradigm, which was later rephrased into “one gene–one polypeptide” (Beadle and Tatum, 1941). Subsequently, first Oswald Avery and then Alfred D. Hershey and Martha Chase made the association between DNA and genetic material through their studies on bacteriophages (Avery et al., 1944; Hershey, 1955). However, the “genetic-biochemical” conception of the gene had its turning point in 1952, when Rosalind Franklin and Raymond Gosling provided an extremely clear x-ray diffraction of DNA helices. In 1953, James D. Watson and Francis Crick finally uncovered the molecular structure of DNA (Watson and Crick, 1953) and inferred that “*the specific pairing (…) immediately suggests a possible copying mechanism for the genetic material*.” Overall these discoveries led to the realization that the genetic material is made up of a chain of polynucleotides, called DNA, and to the establishment of the central Dogma, which states that polypeptides are translated from RNA which is transcribed from DNA. Together with the Watson-Crick double helix model, the relation between DNA and polypeptide synthesis provided a mechanistic model of gene and gene activity and inaugurated the molecular biology era.

**Figure 2:**
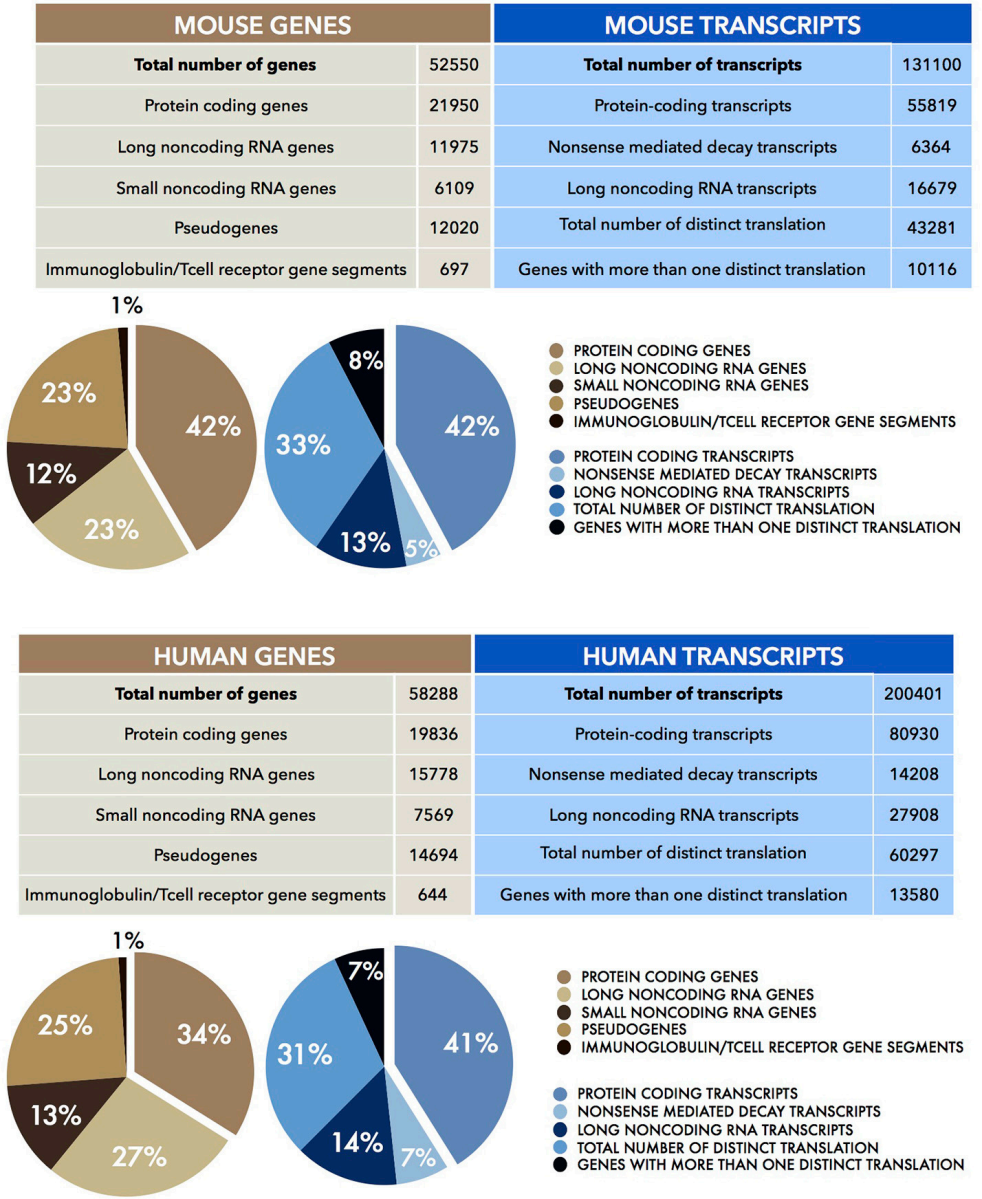
Human and Mouse transcriptomes. The tables show the total number of mouse (UP) and human (DOWN) genes (brown) and transcripts (blue), together with their sub-partition (as percentages, %) in different classes of coding and noncoding RNAs. Data were obtained from the current Mouse (version M15) and Human (version 27) GENCODE datasets (Harrow et al., 2006, 2012; Mudge and Harrow, 2015).

In eukaryotes, the discovery of the diverse modalities of RNA maturation evolved the awareness of colinear relationship of DNA, RNA, and polypeptides. The existence of alternative splicing, 5' and 3'-ends alternative maturation and RNA editing processes allow a single gene to produce multiple proteins by means of a single act of transcription. That only the minority of the transcribed genes in higher eukaryotes encode for proteins suggests that the genome has a potential which extends beyond the discrete coding loci. The rest of the genome (at least 60%) is transcribed independently of its coding capabilities (Berretta and Morillon, 2009; Djebali et al., 2012; Figure 2). These discoveries fuelled debate about the relationship between the number of protein-coding genes and the complexity of the biology of higher organisms (Bickel and Morris, 2006; Mercer and Mattick, 2013). While the number of coding loci remains virtually fixed throughout evolution, the content of noncoding DNA increases. This implies new and as yet unexplored functions for this part of the genome, including the transcription of noncoding RNAs (Rinn and Chang, 2012; Batista and Chang, 2013). This finally changed the concept of the gene from “coding” into “transcriptional unit.”

The concept of the gene is evolving so fast that we are not comfortable giving a more fixed definition to the term other than “*a strategy used by evolution to allow the survival of life*,” for those readers who seek a timeless explanation.

### Zooming out: gene regulation and the “omic” era

If the definition of gene is complex, then the issue “how does a gene work?” is even more arduous to resolve. In their studies on the lactose (Lac) operon of *E. coli*, François Jacob and Jacques Monod provided a first paradigmatic view of genetic (transcriptional) regulation (Jacob and Monod, 1978). The operon discovery marked a crucial point in science, demonstrating that genes do not work as isolated entities. Indeed, although oversimplified, it represents an elegant interpretation of how genes can be regulated in a coordinated fashion in response to environmental conditions.

In the last two decades, many efforts have been made to get a more comprehensive answer to the question, “how do genes work *together*?” A partial answer came when the International Human Genome Sequencing Consortium, in the framework of the Human Genome Project (HGP) provided a more accurate quantification of the number of genes by publishing the complete human genome sequence (Craig Venter et al., 2001; Lander et al., 2001). The quantification of gene loci in the haploid genome was based on the presence of predicted and known Open Reading Frames (ORF). The regions not predicted to be a gene were defined as “junk” DNA (Ohno, 1972; Niu and Jiang, 2013). In the context of the HGP, a large proportion of “junk” DNA emerged unexpectedly as positively selected by evolution and conserved among the human, dog, mouse and other vertebrate genomes (Mouse Genome Sequencing Consortium et al., 2002; Lindblad-Toh et al., 2005). Moreover, a paucity in the number of coding genes emerged. Researchers were surprised to find a figure as low as ~22,300 loci, especially when compared to the 60,000 genes of the single-cell organism *T. vaginalis* (Craig Venter et al., 2001; Lander et al., 2001). It had seemed obvious that humans would have more protein-coding genes than plants. However, this was not the case, as the number of protein-coding genes of *A. thaliana* is approximately the same as that of humans.

These observations suggested that there is more to the genome than protein-coding genes. It anticipated the outcomes of the two main genome analysis projects of the last 15 years, namely FANTOM (Carninci et al., 2005) and ENCODE (Kawai et al., 2001; ENCODE Project Consortium et al., 2007). The aim of the two consortia was to identify and characterize all the functional elements of the mammalian genome and the entire transcriptional landscape. They used multiple sequencing-based approaches such as high throughput cDNA sequencing, Serial Analysis of Gene Expression (SAGE), Cap Analysis Gene Expression (CAGE), Paired End Tags (PET) together with high resolution tiling arrays and Chromatin immunoprecipitation (ChIP) sequencing (Shiraki et al., 2003; Morozova and Marra, 2008). This unprecedented quantity of continuously updated data revealed that the human genome was not a large container of short transcribed sequences interspersed in a genomic desert, but it was rather, pervasively (at least 70%) transcribed in a lattice of transcripts (Figure 2). In addition to the Transcriptional Active Regions (TARs), showing well-defined physical edge, and to alternative transcriptional start sites (TSS), several other genomic regions defined as “regulatory elements” also emerged to be transcribed. In fact, genes appeared to extend into the “intergenic space” giving rise to a plethora of transcripts which numbered five times more than the number of total genes. The transcripts that were not associated with polysomes, thus candidates for a protein-independent function, constituted the class of noncoding RNAs (ncRNAs).

The fact that mammalian genomes are pervasively transcribed is now well accepted. The “junk” DNA interpretation has fallen out of favor, since pervasive transcription and also developmental control of ncRNA expression, together with high promoter conservation, have provided consistent evidence of global functionality (Berretta and Morillon, 2009; Djebali et al., 2012). Indeed, functional studies in knockout mice have provided compelling evidence for the requirement and sufficiency of particular noncoding transcripts for organ development and function (Ripoche et al., 1997; Moseley et al., 2006; Anguera et al., 2011; Nakagawa et al., 2011; Zhang et al., 2012; Sauvageau et al., 2013).

### *Potentiality* and *Actuality*: genome and epigenome

With the exception of lymphocytes, all nucleated human cells contain the same genome. Nevertheless they show very different morphological and functional characteristics depending on cell type, developmental stage, sex, and age. Eric Lander defined the genome as “*a landscape (…) a whole geography of distributions (…) a storybook that's been edited for a couple billion years. And you could take it to bed like A Thousand and One Arabian Nights, and read a different story in the genome every night*.” Thus, each cell of the same individual tells a different story, even if the storybook is the same. This observation can draw on the Aristotelian theory of potentiality and actuality. Potentiality represents the genotype, which contains all the information needed to develop the function, while actuality is the motion of the genotype into a phenotype that is the composite of an organism's observable features. The driver of the switch from potentiality to actuality, however, was unknown. In the late nineteenth century, the question of how a fertilized egg can give rise to a complex organism with cells of varied phenotypes was the object of long debates between two main schools of embryologists. The “pre-formationists,” who thought that each cell contains preformed elements that enlarge during development, while the “epigenesists” thought that chemical reactions among soluble components execute the developmental plan (Felsenfeld, 2014). Indeed, it was by studying the developmental processes that the clear divergence of phenotypes among differentiating cells and tissues and the fact that they are clonally inherited by the dividing cells became evident.

Historically, the term “epigenetics” was introduced by C. Waddington in the 1940s to describe “*the interactions of genes with their environment, which bring the phenotype into being*” (Waddington, 1940). The physical importance of gene position along the chromosomes was first demonstrated in D. melanogaster by Muller in 1930 (Muller, 1930) and defined as “Position-Effect Variegation, PEV.” More generally, these studies addressed the functional differences between two different physical states of the DNA: (i) the heterochromatin, which corresponds to regions of the genome that contain low gene density and is transcriptionally inactive and (ii) the euchromatin, which corresponds to regions with a high density of genes and is transcriptionally active (HSU, 1962). Both euchromatin and heterochromatin are associated with specific DNA methylation and histone modification patterns, leading to the existence of an “epigenetic code” which determines specific chromatin states and, by consequence, gene expression (Felsenfeld, 2014). These modifications involve histone-tail chemical modifications, DNA methylation, histone variants and ATP-remodeling complexes, which are crucial for the establishment of the epigenetic landscape and for the appropriate progression of cell differentiation (Yuan, 2012).

Recent studies have demonstrated that environmental and lifestyle factors may influence epigenetic mechanisms (i.e., DNA methylation, histone acetylation and chromatin plasticity) (Weaver et al., 2004; Feil and Fraga, 2012) and that the acquired modifications can also be transmitted through nonDNA sequence-based (transgenerational) hereditability (Grossniklaus et al., 2013; Szyf, 2013; Dias and Ressler, 2014; Bohacek and Mansuy, 2015; Miska and Ferguson-Smith, 2016). This phenomenon represents a powerful means of change as it allows for the modification of phenotypes without genotype changes. Based on these discoveries, we can now assume two different levels of epigenetic regulation. The first ensures the mitotic inheritance of differentiated cellular states during development (Felsenfeld, 2014), while the second ensures transgenerational inheritance. This occurs through meiosis and acts as an additional evolutionary driving force together with natural selection and genetic drift (Grossniklaus et al., 2013; Miska and Ferguson-Smith, 2016).

## Noncoding RNAs: new players in old processes

### From the “protein centric” view to non-coding RNAs as functional molecules

Understanding of the functional importance of RNA started with the discovery of messenger RNA (mRNA) (Brenner et al., 1961; Jacob and Monod, 1961), ribosomal RNA (rRNA) (Scherrer and Darnell, 1962; Scherrer et al., 1963) and transfer RNA (tRNA) (Hoagland et al., 1958). Other classes of relatively small ncRNAs were later identified and characterized such as the small nuclear RNA (snRNA) (Wassarman and Steitz, 1992), the small nucleolar RNA (snoRNA) (Bachellerie et al., 2002), the piwi-interacting RNA (piRNAs) (Cox et al., 1998), the microRNA (miRNA) (Lee et al., 1993) and the small interfering RNA (siRNA) (Fire et al., 1998; Hamilton and Baulcombe, 1999). The versatility of RNA functions was further emphasized in the 1980s, when Thomas Robert Cech discovered ribozymes and “*established that RNA, like a protein, can act as a catalyst in living cells*” (Kruger et al., 1982). The significance of this work was recognized with the 1989 Nobel Prize in Chemistry.

In the early 1990s the discovery of H19 (Brannan et al., 1990) and *Xist* (Brockdorff et al., 1992; Brown et al., 1992) uncovered the existence of functional long noncoding RNAs (lncRNA) involved in epigenetic regulation. The abundance of this class of noncoding transcripts was revealed by the advent of deep-sequencing approaches. Many common features have been observed between lncRNAs and mRNAs. For instance, lncRNA loci display analogous genetic marks at their regulatory or transcribed regions and are bound by the RNA polymerase II (Pol II). In addition, similarly to mRNAs, lncRNAs contain introns and present a 7-methylguanosine cap at their 5′ end and a poly(A) chain at their 3′ end. Despite their similarities, lncRNAs were primarily considered to be the sub-product of transcriptional noise resulting from low RNA Polymerase fidelity, transcription initiation leakiness (Struhl, 2007) or incidental transcription at enhancer regions (De Santa et al., 2010). This was probably due to their low levels of expression and sequence conservation (Mercer et al., 2011; Nitsche and Stadler, 2017) together with the lack of loss-of-function studies.

LncRNAs display a dynamic pattern of expression in differentiation and development, the specific binding of transcription factors on their promoters, and the presence of peculiar chromatin signatures, such as DNase1 hypersensitivity sites and H3K9ac, H3K4me3, and H3K36me3 histone modifications in cells where they are transcribed (Kawai et al., 2001; Guttman et al., 2009; Rinn and Chang, 2012; Kung et al., 2013). Moreover they show high tissue specificity (Gloss and Dinger, 2016) and their misregulation has been associated with several pathological states (Shi et al., 2013; Tang et al., 2013; Ounzain and Pedrazzini, 2015; Uchida and Dimmeler, 2015; Ballarino et al., 2016).

### Mechanistic examples of long non-coding RNAs

In recent years, most lncRNA experimental biology has been observational. Indeed, while high-throughput sequencing approaches have provided comprehensive catalogs of lncRNA genes and transcripts, a bottleneck exists between the large datasets and poor validation methods. Indeed, to date, only a limited number of lncRNAs have been functionally characterized (Johnsson et al., 2014).

**Figure 3:**
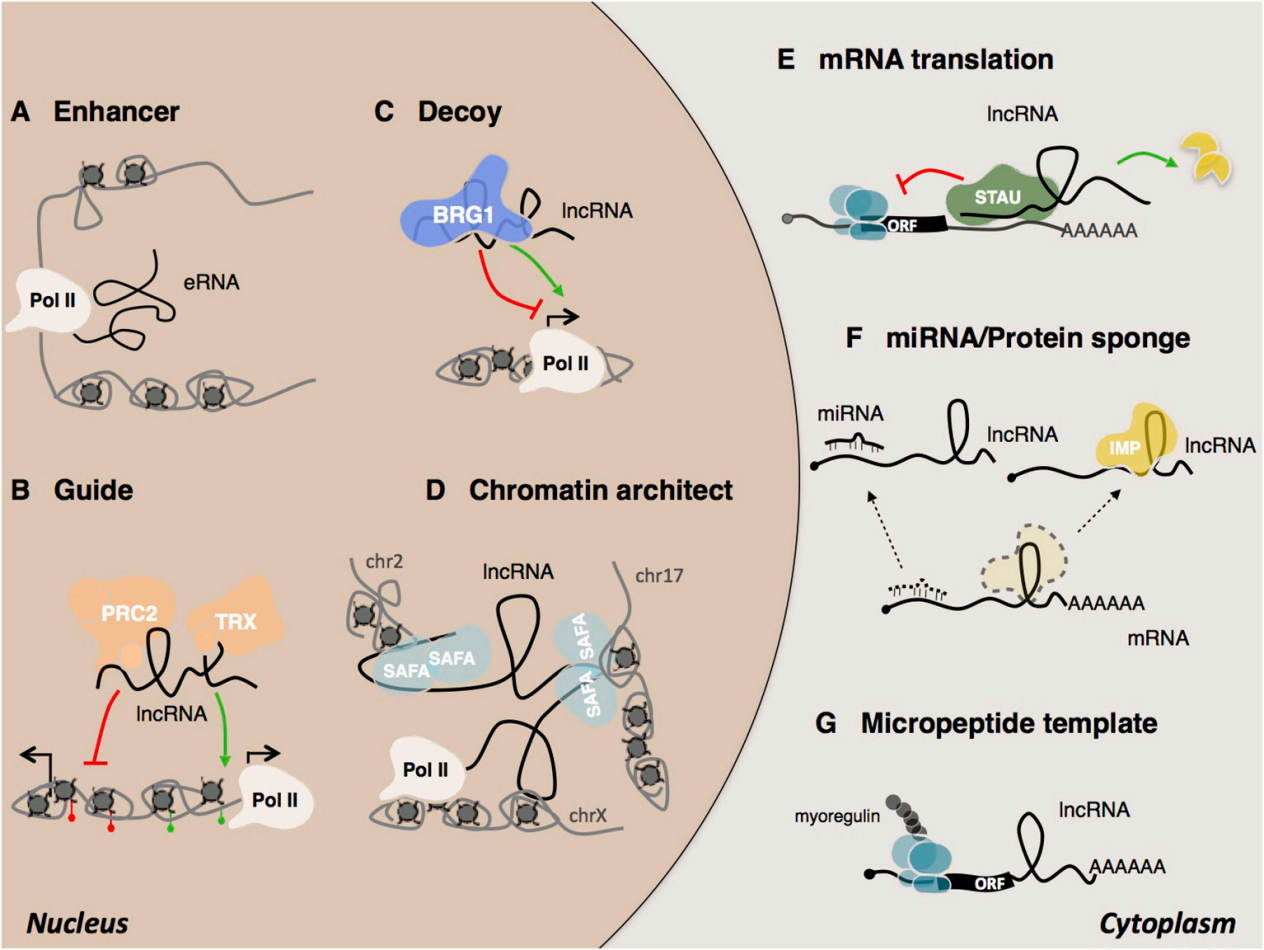
Mechanisms of lncRNA actions. In the nucleus, lncRNAs can act as **(A)** eRNA (enhancer RNA) (Mousavi et al., 2013, 2014; Mueller et al., 2015), **(B)** guide (Rinn et al., 2007), **(C)** decoy (Han et al., 2014) **(D)** chromatin architect (Hacisuleyman et al., 2014). In the cytoplasm, lncRNAs can **(E)** regulate mRNA translation and stability (Gong and Maquat, 2011; Kretz et al., 2013; Wang et al., 2013), **(F)** act as sponges for other transcripts or proteins (Gong et al., 2015), **(G)** serve as micropeptide templates (Anderson et al., 2015; Nelson et al., 2016).

The high-sensitivity interactome techniques developed in recent years which have made it possible to map RNA/RNA RNA/protein RNA/DNA interactions (Kashi et al., 2016). Thanks also to recently developed computational tools (Huarte et al., 2010; Agostini et al., 2013; Cheng et al., 2015; Suresh et al., 2015; Ribeiro et al., 2017), several examples of lncRNA mode of action have started to emerge (Figure 3). LncRNAs can be detected in the nucleus, cytoplasm, or both. Cytoplasmic lncRNAs usually act at post-transcriptional level by regulating the stability and/or the translation of target mRNAs. Thus, both BACE1 AS (Faghihi et al., 2008) and TINCR (Kretz et al., 2013) have been shown to increase the stability of their target RNAs while, the group of cytoplasmic 1/2sbsRNAs, act in the opposite way by favoring STAU1-mediated decay *via* Alu elements (Gong and Maquat, 2011; Wang et al., 2013). An alternative mode of action regards the regulation of mRNA translation by means of complementary base pairing. Examples include the regulation mediated by Uchl1as1 (Carrieri et al., 2012) and p21 (Yoon et al., 2012) lncRNAs. Cytoplasmic lncRNAs can also act as competing endogenous RNAs (ceRNAs) (Cesana et al., 2011) and function as “miRNA sponges” to protect the target mRNAs from repression. Recently, an additional example of ceRNA was found in the newly identified class of circular RNAs (circRNAs). This is the case of the circular ciRS-7 transcript, which contains more than 70 selectively conserved miR-7 target sites (Hansen et al., 2013; Memczak et al., 2013; Piwecka et al., 2017). Despite their common ribosome occupancy (Guttman et al., 2013), lncRNAs are defined as transcripts that do not encompass translation. However, two recent examples of lncRNAs that encode for short and functional micropeptides have been described (Anderson et al., 2015; Nelson et al., 2016) suggesting a coding-based mechanism of action for these ncRNAs.

Although several archetypes of cytoplasmic species have been described, most lncRNAs are predominantly found to be enriched in the nucleus and in particular associated with chromatin (Derrien et al., 2012). This observation supports the idea that many lncRNAs are engaged in the epigenetic and transcriptional regulation of gene expression (Fatica and Bozzoni, 2014; Morlando et al., 2014). Also in the case of nuclear lncRNAs, some common modes of action have emerged and been classified by the scientific community (Kung et al., 2013; Fatica and Bozzoni, 2014; Kashi et al., 2016). They may work either in *cis*, when they act in the vicinity of their transcriptional locus or in *trans*, when they act at a distance in the regulation of intra or inter chromosomal loci (Rinn and Chang, 2012). Recognition of the target regions by lncRNAs can occur through different mechanisms such as bridging proteins, RNA-RNA or RNA-DNA hybrids, including triple helix or R-loop formation (Engreitz et al., 2016a). The ability of lncRNAs to act as scaffold molecules allows them to interact simultaneously with several molecular components and chromatin remodeling complexes (Tsai et al., 2010). As guide molecules, they have the ability to recruit functional protein complexes to conduct them directly to specific target loci (Lanz et al., 1999; Rinn et al., 2007; Kino et al., 2010; Di Ruscio et al., 2013). LncRNAs can also influence their targets indirectly. For instance, as decoy molecules they can change the availability of transcription factors and as a consequence, reduce DNA binding capacity (Wang et al., 2008; Kino et al., 2010; Guttman and Rinn, 2012; Rinn and Chang, 2012; Batista and Chang, 2013). The participation of lncRNAs in the formation of nuclear domains has long been the subject of speculation (Nickerson et al., 1989; He et al., 1990). Today several lncRNAs have been shown to act as chromosomal architects for the spatial coordination of gene expression (Korostowski et al., 2012; Hacisuleyman et al., 2014; Zhang H. et al., 2014; Engreitz et al., 2016b). Examples include *Xist* and FIRRE. *Xist* promotes X inactivation by acting on the three-dimensional organization of the X chromosome (Splinter et al., 2011; Engreitz et al., 2013; Giorgetti et al., 2016). Another example is the lncRNA FIRRE, which controls murine adipogenesis by promoting the formation of inter-chromosomal domains among functionally related genes (Hacisuleyman et al., 2014).

A widely debated mechanism of nuclear lncRNA action concerns their ability to function in the recruitment of chromatin modifying complexes for the regulation of chromatin states. A paradigmatic example is represented by HOTAIR (Rinn et al., 2007), which has been demonstrated to act as a scaffolding molecule, capable of interacting simultaneously with the PRC2 silencing complex at its 5'-end and with the LSD1/CoREST/REST complex at its 3'-end (Tsai et al., 2010). Similarly to HOTAIR (Rinn et al., 2007) also *Xist* (Zhao et al., 2008), Bvht (Klattenhoff et al., 2013), Kcnq1ot1 (Pandey et al., 2008), FENDRR (Grote et al., 2013), CARMEN (Ounzain et al., 2015), and Chaer (Wang et al., 2016) represent other examples of lncRNAs where the interaction with PRC2 has been proposed.

A more systematic search has revealed that a vast number of transcripts interact with PRC2 and that siRNA mediated depletion of certain lncRNAs associated with PRC2 leads to changes in gene expression (Khalil et al., 2009; Davidovich et al., 2013; Kaneko et al., 2013; Beltran et al., 2016). However the functional interaction between PRC2 and lncRNAs is still under debate. In particular two recently published papers (Amândio et al., 2016; Blanco and Guttman, 2017; Portoso et al., 2017) have demonstrated that the binding with the PRC2 complex is dispensable for HOTAIR function. This evidence has raised a number of concerns regarding the specificity and the functional relevance of this interaction (Blanco and Guttman, 2017). A second example is represented by *Xist*, which is known to coordinate X chromosome inactivation (XCI) by silencing transcription through the recruitment of several chromatin modifying complexes, including PRC1 and PRC2 (Wutz et al., 2002; Schoeftner et al., 2006; Zhao et al., 2008; Wutz, 2011; Almeida et al., 2017; Pintacuda et al., 2017). However, doubt was recently cast on this paradigm of epigenetic regulation following a number of studies showing that ablation of different PRC2 components has no impact on *Xist*-mediated transcriptional silencing (Kalantry and Magnuson, 2006; Schoeftner et al., 2006). On the other hand, the deletion of the A-repeat region of *Xist*, which is required for *Xist*-mediated transcriptional silencing (Wutz et al., 2002; Zhao et al., 2008), does not preclude PRC2 recruitment to the X chromosome (Plath et al., 2003; da Rocha et al., 2014). Moreover, several observations also argue against a direct interaction between *Xist* RNA and PRC2 proteins (Cerase et al., 2014). The indication supporting the broad lncRNA binding with PCR2 has also recently been revisited by the latest discoveries from Guttman's lab, which show that the purification of the *Xist* lncRNA through UV-crosslinking-based strategies failed to identify its direct interaction with PRC2 (McHugh et al., 2015). Overall, this evidence has brought into question the requirement of PRC2 for lncRNA functioning. It suggests that there may as yet be unknown mechanisms of gene regulation *in vivo*, possibly acting without the need for their close proximity. Finally, in line with the high functional heterogeneity of lncRNAs, a completely novel mechanism of regulation has been proposed by Olson's lab which holds that the action of the Hand2-associated Uph lncRNA transcription, and not the transcript itself, is the only functional requirement for gene regulation and heart development (Anderson et al., 2016).

The aforementioned examples represent only the tip of a regulatory RNA iceberg in which lncRNAs together with their interactors are involved. What clearly emerges from these studies is the vagueness of some of the mechanisms proposed, which highlight the need to develop more reliable techniques to better define the *bona fide* interactions that occur in living cells. Although the molecular comprehension of these noncoding mechanisms of action has improved considerably in recent years, significant efforts are still required.

## New technologies for the study of lncRNAs/protein interactions

Because of the existence of a wide spectrum of transcripts, RNA-protein interactions represent a conspicuous part of the interactome and fully understanding them is among the most ambitious goals of RNA researchers. Many variables have been shown to control the interactions between RNA and proteins. These include the binding affinity of the protein for the RNA substrate, the concentration of the respective binders, and the competition with other RNAs and/or proteins (Jankowsky and Harris, 2015). Moreover, as specific sets of RNAs and proteins can be localized to defined areas, also subcellular localization and compartmentalization can influence the occurrence of interactions. All together, these variables establish the homeostatic equilibrium on which the biological complexity is established.

Long non-coding RNAs often exert their functions by binding one or more proteins. Hence, the identification of lncRNA-proteome contacts is a crucial step toward the understanding of the functional mechanisms in which these noncoding molecules act. Several lncRNAs have been shown to form complexes with proteins in both the nuclear and the cytoplasmic compartments (Fatica and Bozzoni, 2014). Thus, it would be reasonable to assume that other RNA-protein interactions might form similar complexes that affect biological functions. Despite their biological importance, RNA-protein interactions are much less well characterized than those for DNA-protein complexes.

Several computational algorithms have been developed to predict the interaction probability of a particular RNA-protein pair by taking advantage of the structural information obtained experimentally and mostly available on the Protein Data Bank (PDB) databases (Berman et al., 2000). These algorithms have been applied to create computational tools and web servers to predict RNA/protein interaction partners. A detailed description of these tools is beyond the scope of this review but those readers who seek more insights will find it useful to read the manuscript from the Dobbs' lab (Muppirala et al., 2013) and book chapters (Chang, 2012; Meller and Porollo, 2012). More recently, an innovative large-scale pipeline has been developed by Ribeiro and colleagues to identify candidate lncRNAs acting as scaffolding molecules for protein complexes (Ribeiro et al., 2017). By using a catRAPID-based omics algorithm (Agostini et al., 2013), these authors have predicted a number of 847 lncRNAs (~5% of the lncRNA transcriptome) capable of scaffolding half of the known protein complexes and network modules (Ribeiro et al., 2017). This suggests, for the first time, that the lncRNA-mediated scaffolding of protein complexes and modules is a common mechanism in human cells.

**Figure 4:**
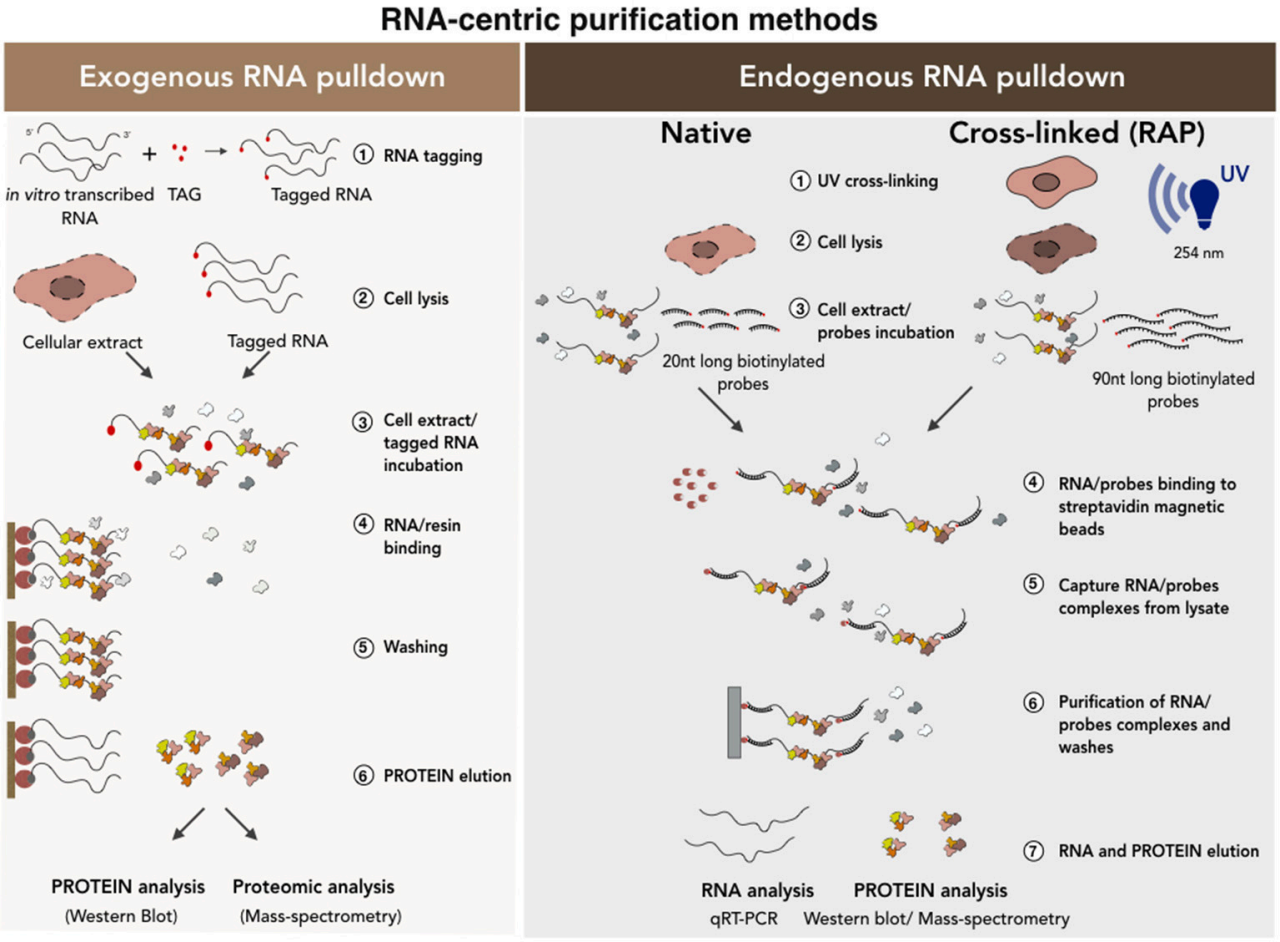
RNA-centric purification methods for the analysis of the RNA-protein interactions. A schematic of exogenous (Left) vs. endogenous (Right) RNA pull-down methods. In the exogenous RNA pull-down the transcript of interest is tagged (i.e., biotinylation) by *in vitro* transcription. The co-purified proteins are collected and analyzed by Western Blot or Mass-spectrometry analyses. Endogenous RNA pull-down uses native or cross-linked conditions. The experimental procedure is conceptually the same except that, in the case of the cross-linked approach, a set of 90 nucleotides (90 nt) long biotinylated probes is used. See text for further details.

**Figure 5:**
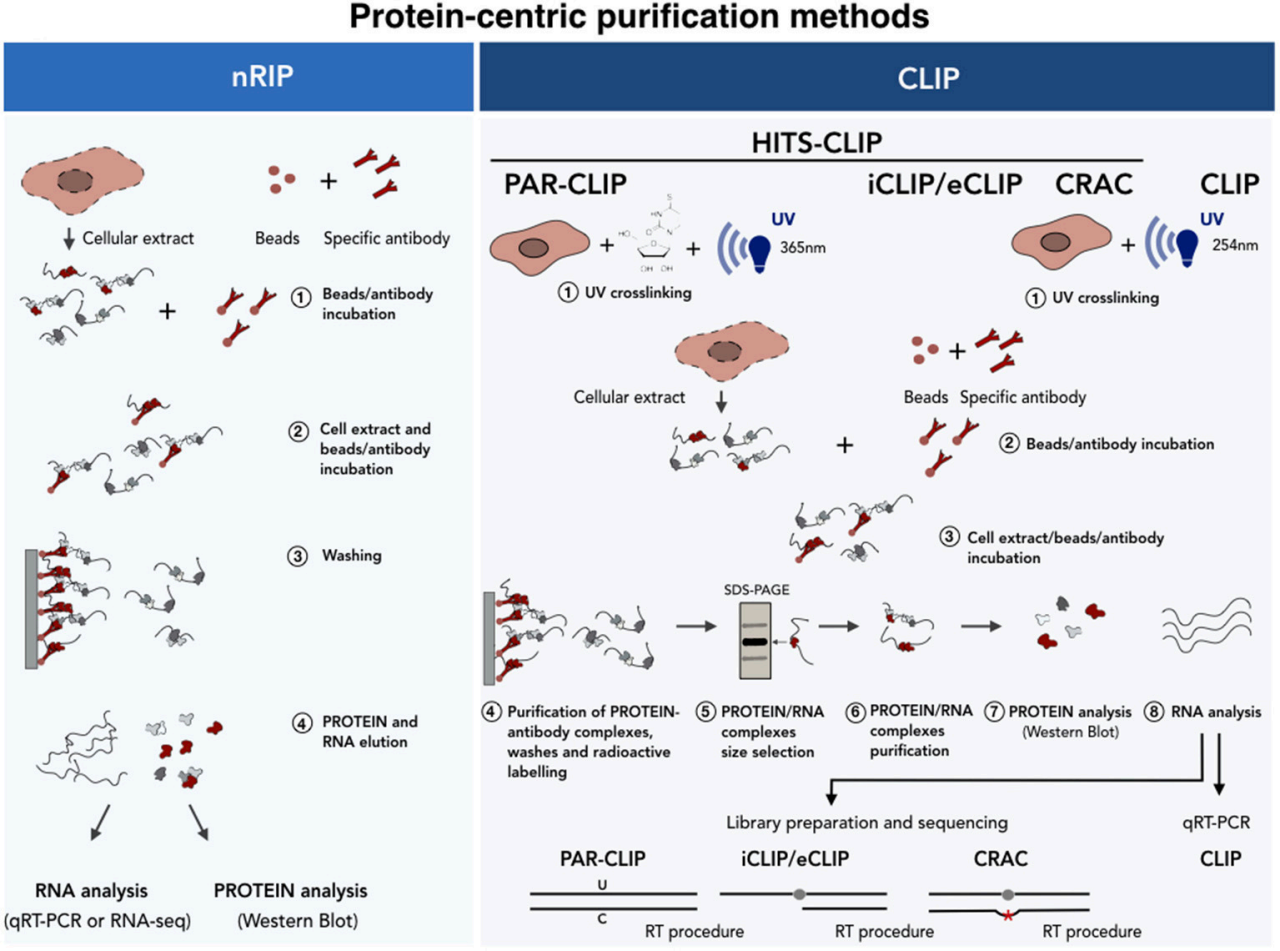
Protein-centric purification methods for the analysis of the RNA-protein interactions. A schematic of the native (Left) vs. cross-linked (Right) RNA immunoprecipitation methods. In the case of nRIP the co-purified RNA is analyzed by qRT-PCR or RNA-seq analyses. In HITS-CLIP the protein-RNA complexes are isolated by SDS-PAGE-based size selection before sequencing. In PAR-CLIP cells cultured in the presence of nucleotide analogs (i.e., 4-thiouridine), which undergo U-C transition upon UV, are used as starting material. iCLIP uses the reverse transcriptase arrest induced by the crosslinked protein. Finally, CRAC relies on mutational analysis induced by reverse transcription at cross-linking sites. See text for further details.

Despite the huge potential of these computational methods, experimental validation is essential to verify the occurrence of the predicted interactions *in vivo*. These experimental approaches can be classified into two main categories, namely RNA-centric or protein-centric methods (Figures 4, 5). The first approach relies on the ability to isolate and purify a specific RNA to subsequently check for the interacting proteins (Lingner and Cech, 1996; Hogg and Collins, 2007; Rinn et al., 2007; Klattenhoff et al., 2013; Yang et al., 2014; McHugh et al., 2015; Wang et al., 2016). In contrast, the protein-centric techniques are based on the immunoprecipitation of a specific protein followed by the analysis of co-precipitated RNAs (Mili, 2004; Wang et al., 2009; Haecker and Renne, 2014). A challenging point that needs discussion consists in the ability of such methods to discriminate between *in vivo* (in the cells) and *in vitro* occurring interactions (Jankowsky and Harris, 2015). For this reason, many crosslinked-based methods have been recently developed. These can be used in alternative to the native approaches to reduce the possibility that nonphysiological background interactions can occur *in vitro* during the experimental procedures (Kalantry and Magnuson, 2006; Xue et al., 2016; Portoso et al., 2017).

### RNA-centric purification methods

There are two main categories of RNA-centric purification methods (Figure 4): (i) the exogenous RNA pull-down, which based on *in vitro* RNA affinity capture methods and (ii) the endogenous RNA pull-down, which is based on the purification of the endogenous transcript under native or ultraviolet (UV) cross-linking conditions. In the first method (Figure 4, left), the candidate RNA is transcribed *in vitro* as fused to an aptamer and then incubated with nuclear, cytoplasmic or total protein extracts. The newly-formed RNA-protein complexes are purified on a solid support or resins containing proteins or different organic molecules depending on the tagging strategy used (i.e., streptavidin, MS2 viral coat protein) (Bardwell and Wickens, 1990; Slobodin and Gerst, 2010). Finally, aspecific binders are removed from the solid support following stringent washes and the RNA-protein complexes eluted by boiling in sodium dodecyl sulfate (SDS)-containing buffers (Srisawat and Engelke, 2001; Carey et al., 2002; Huarte et al., 2010; Lee et al., 2013; Leppek and Stoecklin, 2014; Liu et al., 2017). This method makes the study of transcripts with low expression possible because it favors protein yield. However, the use of a synthetic RNA to capture proteins and the fact that the binding between RNAs and proteins occur *in vitro*, can lead to possible artificial interactions. In addition, RNA folding was shown to be regulated and strongly influenced by cellular environment and by the interaction with specific chaperon proteins (Schroeder et al., 2002). As the function of RNA is deeply influenced by its structure and since the *in vitro* folding may be significantly different from the *in vivo* conditions (Schroeder et al., 2002; Fallmann et al., 2017; Leamy et al., 2017), potential RNA misfolding occurring *in vitro* can interfere with results reliability. For all these reasons, data need to be carefully checked and the use of alternative methods is strongly recommended.

The native endogenous RNA pull-down (Figure 4, right) is based on the purification of the endogenous RNA transcript by using a set of antisense biotinylated probes. This set of probes is incubated with the cellular extract in specific buffer conditions that allow their base-pairing with the RNA target. The mixture is then incubated with streptavidin-coated magnetic beads which specifically bind the biotinylated oligos-target RNA-protein complex allowing its precipitation (Zielinski et al., 2006; Tsai et al., 2011; Legnini et al., 2014; McHugh et al., 2015; Ribeiro et al., 2017). The beads are finally washed and the precipitated RNA-protein complexes analyzed. In comparison to the previous method, here the physiological RNA-protein interactions occurring *in vivo* are better preserved. However, also in this case, the occurrence of background interactions cannot be excluded (Portoso et al., 2017). Furthermore, if the levels of the target RNA in the cell are low, its purification can be more challenging. Finally, as RNA is not a linear molecule and can be engaged in some parts in protein binding, a wide range of probes covering the entire sequence must be tested in order to find free regions available to probes.

The inability to discriminate between specific and contaminating interactions constitutes the weakest point of the above-mentioned methods. In order to identify only the physical contacts that occur *in vivo*, the endogenous RNA pull-down can be implemented by the addition of an ultraviolet crosslinking step followed by denaturing (up to 8M of urea) washing conditions (Baltz et al., 2012; McHugh et al., 2015). This approach makes it possible to discard all the contaminating RNA binding proteins that can be abundant in native purifications. This is because the use of short UV light wavelengths (usually 254 nm) induces the formation of covalent bonds only between RNA and interecting proteins (Zeng et al., 2006; Baltz et al., 2012).

The choice of a negative control constitutes an important step for ensuring robustness to the results. The use of an antisense RNA is considered the best negative control for the exogenous RNA pull-down (Rinn et al., 2007; Klattenhoff et al., 2013). In the endogenous RNA pull-down the negative control consists of a set of probes that do not target any endogenously expressed RNA sequence (Legnini et al., 2014; Ribeiro et al., 2017). In the case of the cross-linked endogenous RNA pull-down, the ideal negative control would be a noncrosslinked sample and/or a specific RNA whose interactors are known (Baltz et al., 2012; McHugh et al., 2015).

### Protein-centric purification methods

Protein-centric methods represent a complementary approach and consist of the immunoprecipitation of the protein of interest with specific antibodies followed by the analysis of co-precipitated RNAs (Figure 5). In recent years two main protein-centric methods have been developed (Peritz et al., 2006; Wang et al., 2009; Haecker and Renne, 2014), namely the native RNA ImmunoPrecipitation (nRIP) (Figure 5, left) and the crosslinking immunoprecipitation (CLIP) (Figure 5, right) approaches. The first approach is usually performed in native conditions (Peritz et al., 2006) and enables the purification of those RNAs which are stably associated, in their natural condition, with the protein complexes. The analysis of co-precipitated RNAs is then performed by quantitative realtime PCR (qPCR) or by RNA-seq. Examples of lncRNAs interactors identified by nRIP are represented by FENDRR (Grote et al., 2013), HOTTIP (Wang et al., 2011) *Mhrt*, (Han et al., 2014), CARMEN (Wang et al., 2011; Grote et al., 2013; Han et al., 2014; Ounzain et al., 2015). In spite of its wide use, nRIP presents a number of limitations and the incidence of contaminating contacts can always occur upon cell lysis (Mili, 2004; Schoeftner et al., 2006; Engreitz et al., 2016b; Blanco and Guttman, 2017). As regards for the RNA-centric methods, false positive contacts can be significantly reduced by the use of short (254 nm) UV irradiation wavelengths, which is the principle of the (UV) Cross-Linked ImmunoPrecipitation (CLIP) approach. It differs from nRIP as, once covalently bound, the protein of interest is immunoprecipitated under stringent conditions after a partial RNAase digestion. The protein-RNA complexes are indirectly labeled by γATP incorporation, denaturated in SDS-containing buffers, size selected by electrophoresis and transferred to nitrocellulose membranes. Proteins are then digested with proteinase K and the recovered RNA ligated to an adapter, reverse transcribed and analyzed by qRT-PCR (Ule et al., 2003).

Since 2003, when the first CLIP approach was used on brain tissues (Ule et al., 2003), many variants of CLIP-based approaches have been developed which combine CLIP experiments with high-throughput sequencing (HITS)-CLIP (also known as CLIP-seq) (Licatalosi et al., 2008; Chi et al., 2009; Guil et al., 2012). Since the determination of the exact RNA/protein binding site is crucial, some upgraded protocols have been developed which enable mapping of the contact region at single nucleotide resolution. The Photo Activatable Ribonucleotide-enhanced (PAR) CLIP (Spitzer et al., 2014) takes advantage of U to C transition induced by UV crosslinking (365 nm) after the incorporation of a nucleotide analog (i.e., 4'-thiouracil) which is provided during cell culturing. The weakness of this technique is that it cannot be applied to primary tissues since the incorporation of nucleotide analogs occurs during cell replication. The identification of binding sites can also be achieved by the use of alternative CLIP approaches, such as crosslinking and analysis of cDNA (CRAC) (Bohnsack et al., 2012) and the individual-nucleotide resolution UV crosslinking and immunoprecipitation (iCLIP) (König et al., 2010; Huppertz et al., 2014). In these approaches, the binding of the protein to the RNA causes occasional reverse transcription arrest (iCLIP), or transcription errors (deletions or substitutions) (CRAC) which makes it possible to map the protein/RNA binding site at single nucleotide resolution upon sequencing.

CLIP-seq approaches are technically demanding and can produce many sequencing artifacts. In order to increase the library generation efficiency thus enhancing the discovery of *bona fide* binding sites, an enhanced-CLIP protocol (eCLIP) has recently been developed. In iCLIP experiments, occasional transcription arrests are mapped through circular ligation based methods. Since this step is often inefficient, Van Nostrand and collaborators have modified this part with the addition of two different adapters in two separate steps. The 3' RNA adapter is ligated directly to the crosslinked RNA after immunoprecipitation, while the 3′ single-stranded DNA adapter is ligated after reverse transcription to the 3'end of the cDNA (Van Nostrand et al., 2016). These modifications are able to maintain the high-resolution of iCLIP increasing at the same time the efficiency of the adapters ligation. This consequently results in an improvement of the library preparation of the purified RNA fragments, resulting in the enhanced technical and biological reproducibility.

Finally, in order to share the huge amount of data produced by these experiments, many CLIP-seq database are now available. These represent a powerful tool for the identification of this type of interactions, taking advantage of the CLIP sequencing data published by the scientific community (Yang et al., 2015). In addition to CLIP experiment datasets, in the recent years other bioinformatics tools have been developed that allow to explore miRNA/mRNA, RNA-RNA, RNA/Protein physical interactions (Yang et al., 2011; Anders et al., 2012; Zhang X. et al., 2014).

## Concluding remarks

In eukaryotes, the expression of genes is controlled at different stages, from the chromatin accessibility of DNA to the RNA transcription, processing and translation. Evolutionary pressure selected a large variety of regulatory means which act to fine-tune gene expression through sophisticated RNA and protein machineries. Overall these processes helped to explain some of the differences observed among different species with relatively similar number of genes. The advent of large-scale analyses of mammalian transcriptomes expanded these regulatory options and revealed that the transcriptional landscape of all organisms is far more intricate than initially imagined. A great part of the genome is pervasively transcribed into a diverse collection of RNAs. These can be divided into the following categories: protein coding (mRNAs), structural (i.e., rRNAs, snRNAs, snoRNAs) and regulatory (i.e., miRNAs, lncRNAs, circRNAs) RNAs.

**Figure 6:**
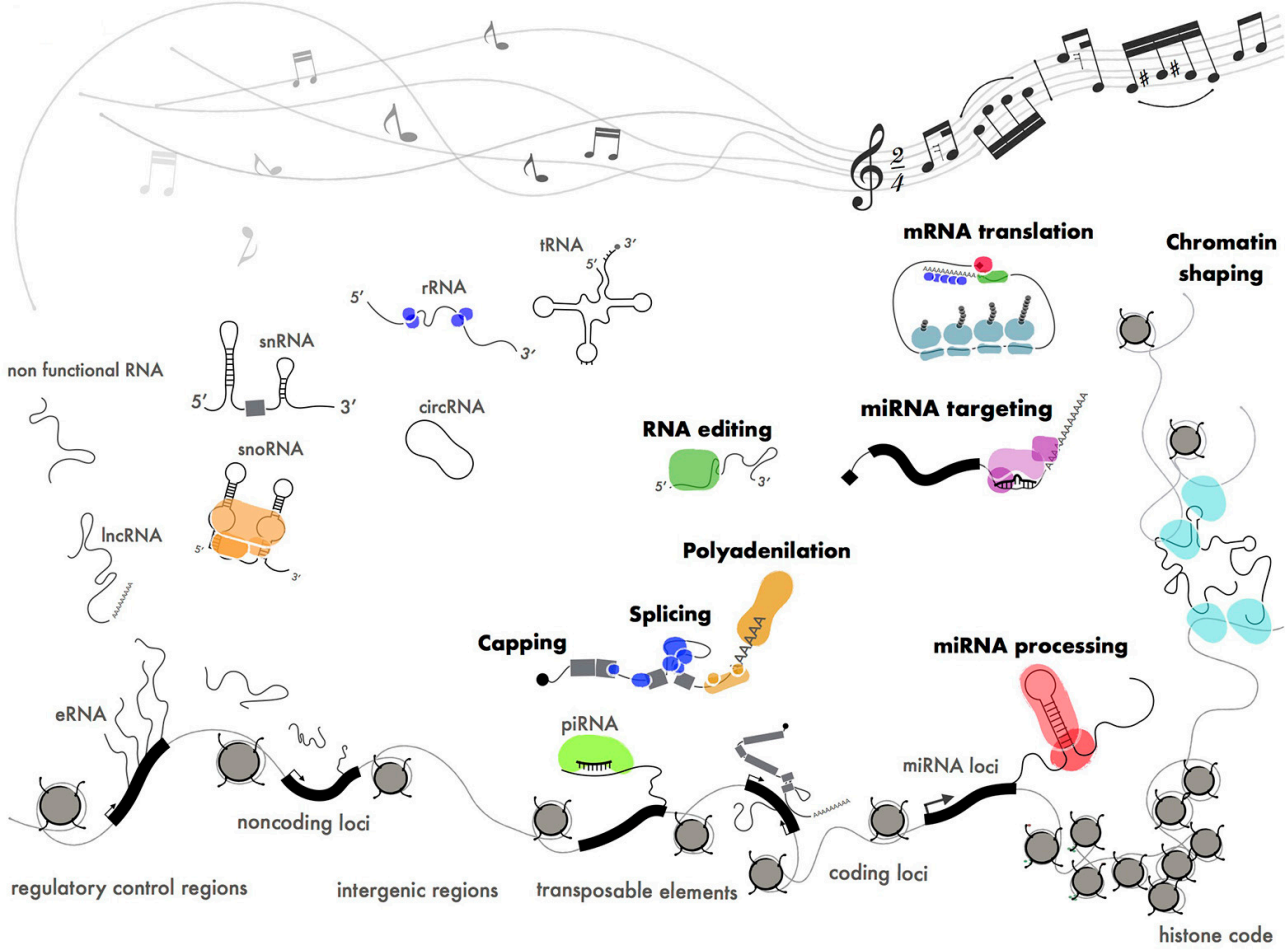
Functional ribonucleoproteins orchestrating gene expression. Different classes of coding (mRNA), structural (tRNA, rRNA, snRNA, snoRNA) and regulatory (miRNA, piRNA, lncRNA) RNAs produced by pervasive transcription. Some of these transcripts have been positively selected by evolution and turned into functional molecules through interaction with protein machineries. The pentagram shows a fragment of Paganini's “Caprice” no. 24.

LncRNAs represent the most recently discovered class of regulatory RNAs which exert their roles through a variety of mechanisms without being translated into proteins. The interaction with proteins enables RNAs to operate through distinct means and to exert a wide-range of functions across diverse biological processes (Figure 6). One intriguing aspect of lncRNAs is their modular structure and their capability to act as scaffold to facilitate different molecular interactions. Thus, mapping the lncRNA-protein contacts remains one of the most significant challenges to understanding their biological roles more deeply. However, despite great progress in the interactomic field, the ability to discriminate between false positive and true interactors is still a significant challenge that needs to be addressed in order to increase the efficiency and the reproducibility of the different approaches. The use of complementary approaches and multiple replicates still constitute the best strategy for validation and enhances the robustness of the interactions identified.

In the attempt to find order within the RNA landscape we could define pervasive transcription as a cocktail of disordered sounds that are selected by evolution to be turned into music (Figure 6). This is in perfect keeping with the Dawkins' “selfish gene” theory (Wade and Dawkins, 1978) where the “gene,” whatever this means, is considered as the unique substrate for selective pressure throughout evolution.

## Author contributions

AC wrote the manuscript and selected the literature. MB proposed the topic, wrote the manuscript and reviewed the text.

### Conflict of interest statement

The authors declare that the research was conducted in the absence of any commercial or financial relationships that could be construed as a potential conflict of interest.

## References

[B1] Agostini F. Zanzoni A. Klus P. Marchese D. Cirillo D. Tartaglia G. G. (2013). catRAPID omics: a web server for large-scale prediction of protein-RNA interactions. Bioinformatics 29, 2928–2930. 10.1093/bioinformatics/btt495 23975767 PMC3810848

[B2] Almeida M. Pintacuda G. Masui O. Koseki Y. Gdula M. Cerase A.. (2017). PCGF3/5-PRC1 initiates Polycomb recruitment in X chromosome inactivation. Science 356, 1081–1084. 10.1126/science.aal2512 28596365 PMC6522364

[B3] Amândio A. R. Necsulea A. Joye E. Mascrez B. Duboule D. (2016). Hotair is dispensible for mouse development. PLoS Genet. 12:e1006232. 10.1371/journal.pgen.1006232 27977683 PMC5157951

[B4] Anders G. Mackowiak S. D. Jens M. Maaskola J. Kuntzagk A. Rajewsky N.. (2012). doRiNA: a database of RNA interactions in post-transcriptional regulation. Nucleic Acids Res. 40, D180–D186. 10.1093/nar/gkr1007 22086949 PMC3245013

[B5] Anderson D. M. Anderson K. M. Chang C. L. Makarewich C. A. Nelson B. R. McAnally J. R.. (2015). A micropeptide encoded by a putative long noncoding RNA regulates muscle performance. Cell 160, 595–606. 10.1016/j.cell.2015.01.009 25640239 PMC4356254

[B6] Anderson K. M. Anderson D. M. McAnally J. R. Shelton J. M. Bassel-Duby R. Olson E. N. (2016). Transcription of the non-coding RNA upperhand controls Hand2 expression and heart development. Nature 539, 433–436. 10.1038/nature20128 27783597 PMC5261552

[B7] Anguera M. C. Ma W. Clift D. Namekawa S. Kelleher R. J. Lee J. T. (2011). Tsx produces a long noncoding RNA and has general functions in the germline, stem cells, and brain. PLoS Genet. 7:e1002248. 10.1371/journal.pgen.1002248 21912526 PMC3164691

[B8] Avery O. T. Macleod C. M. McCarty M. (1944). Studies on the chemical nature of the substance inducing transformation of pneumococcal types: induction of transformation by a desoxyribonucleic acid fraction isolated from pneumococcus Type III. J. Exp. Med. 79, 137–158. 10.1084/jem.79.2.137 19871359 PMC2135445

[B9] Bachellerie J. P. Cavaillé J. Hüttenhofer A. (2002). The expanding snoRNA world. Biochimie 84, 775–790. 10.1016/S0300-9084(02)01402-5 12457565

[B10] Ballarino M. Morlando M. Fatica A. Bozzoni I. (2016). Non-coding RNAs in muscle differentiation and musculoskeletal disease. J. Clin. Invest. 126, 2021–2030. 10.1172/JCI84419 27249675 PMC4887180

[B11] Baltz A. G. Munschauer M. Schwanhäusser B. Vasile A. Murakawa Y. Schueler M.. (2012). The mRNA-bound proteome and its global occupancy profile on protein-coding transcripts. Mol. Cell 46, 674–690. 10.1016/j.molcel.2012.05.021 22681889

[B12] Bardwell V. J. Wickens M. (1990). Purification of RNA and RNA-protein complexes by an R17 coat protein affinity method. Nucleic Acids Res. 18, 6587–6594. 10.1093/nar/18.22.6587 1701242 PMC332614

[B13] Batista P. J. Chang H. Y. (2013). Long noncoding RNAs: cellular address codes in development and disease. Cell 152, 1298–1307. 10.1016/j.cell.2013.02.012 23498938 PMC3651923

[B14] Beadle G. W. Tatum E. L. (1941). Genetic control of biochemical reactions in neurospora. Proc. Natl. Acad. Sci. U.S.A. 27, 499–506. 10.1073/pnas.27.11.499 16588492 PMC1078370

[B15] Beltran M. Yates C. M. Skalska L. Dawson M. Reis F. P. Viiri K. (2016). The interaction of PRC2 with RNA or chromatin is mutually antagonistic. Genome Res. 26, 896–907. 10.1101/gr.197632.115 27197219 PMC4937559

[B16] Berman H. M. Westbrook J. Feng Z. Gilliland G. Bhat T. N. Weissig H. (2000). The protein data bank. Nucleic Acids Res. 28, 235–242. 10.1093/nar/28.1.235 10592235 PMC102472

[B17] Berretta J. Morillon A. (2009). Pervasive transcription constitutes a new level of eukaryotic genome regulation. EMBO Rep. 10, 973–982. 10.1038/embor.2009.181 19680288 PMC2750061

[B18] Bickel K. S. Morris D. R. (2006). Silencing the transcriptome's dark matter: mechanisms for suppressing translation of intergenic transcripts. Mol. Cell 22, 309–316. 10.1016/j.molcel.2006.04.010 16678103

[B19] Blanco M. R. Guttman M. (2017). Re-evaluating the foundations of lncRNA-Polycomb function. EMBO J. 36, 964–966. 10.15252/embj.201796796 28298433 PMC5391134

[B20] Bohacek J. Mansuy I. M. (2015). Molecular insights into transgenerational non-genetic inheritance of acquired behaviours. Nat. Rev. Genet. 16, 641–652. 10.1038/nrg3964 26416311

[B21] Bohnsack M. T. Tollervey D. Granneman S. (2012). Identification of RNA helicase target sites by UV cross-linking and analysis of cDNA. Meth. Enzymol. 511, 275–288. 10.1016/B978-0-12-396546-2.00013-9 22713325

[B22] Brannan C. I. Dees E. C. Ingram R. S. Tilghman S. M. (1990). The product of the H19 gene may function as an RNA. Mol. Cell. Biol. 10, 28–36. 10.1128/MCB.10.1.28 1688465 PMC360709

[B23] Brenner S. Jacob F. Meselson M. (1961). An unstable intermediate carrying information from genes to ribosomes for protein synthesis. Nature 190, 576–581. 10.1038/190576a0 20446365

[B24] Brockdorff N. Ashworth A. Kay G. F. McCabe V. M. Norris D. P. Cooper P. J.. (1992). The product of the mouse Xist gene is a 15 kb inactive X-specific transcript containing no conserved ORF and located in the nucleus. Cell 71, 515–526. 10.1016/0092-8674(92)90519-I 1423610

[B25] Brown C. J. Hendrich B. D. Rupert J. L. Lafrenière R. G. Xing Y. Lawrence J.. (1992). The human XIST gene: analysis of a 17 kb inactive X-specific RNA that contains conserved repeats and is highly localized within the nucleus. Cell 71, 527–542. 10.1016/0092-8674(92)90520-M 1423611

[B26] Carey J. Cameron V. de Haseth P. L. Uhlenbeck O. C. (2002). Sequence-specific interaction of R17 coat protein with its ribonucleic acid binding site. Biochemistry 22, 2601–2610. 10.1021/bi00280a002 6347247

[B27] Carninci P. Kasukawa T. Katayama S. Gough J. Frith M. C. Maeda N.. (2005). The transcriptional landscape of the mammalian genome. Science 309, 1559–1563. 10.1126/science.1112014 16141072

[B28] Carrieri C. Cimatti L. Biagioli M. Beugnet A. Zucchelli S. Fedele S.. (2012). Long non-coding antisense RNA controls Uchl1 translation through an embedded SINEB2 repeat. Nature 491, 454–457. 10.1038/nature11508 23064229

[B29] Cerase A. Smeets D. Tang Y. A. Gdula M. Kraus F. Spivakov M. (2014). Spatial separation of Xist, R. N. A., and polycomb proteins revealed by superresolution microscopy. Proc. Natl. Acad. Sci. U.S.A. 111, 2235–2240. 10.1073/pnas.1312951111 24469834 PMC3926064

[B30] Cesana M. Cacchiarelli D. Legnini I. Santini T. Sthandier O. Chinappi M.. (2011). A long noncoding RNA controls muscle differentiation by functioning as a competing endogenous RNA. Cell 147, 358–369. 10.1016/j.cell.2011.09.028 22000014 PMC3234495

[B31] Chang T.-H. (2012). Chapter 11 Computational approaches to predict protein interaction, in Protein-Protein Interactions - Computational and Experimental Tools, ed Cai W. (InTech).

[B32] Cheng Z. Zhou S. Guan J. (2015). Computationally predicting protein-RNA interactions using only positive and unlabeled examples. J. Bioinform. Comput. Biol. 13:1541005. 10.1142/S021972001541005X 25790785

[B33] Chi S. W. Zang J. B. Mele A. Darnell R. B. (2009). Argonaute HITS-CLIP decodes microRNA-mRNA interaction maps. Nature 460, 479–486. 10.1038/nature08170 19536157 PMC2733940

[B34] Cox D. N. Chao A. Baker J. Chang L. Qiao D. Lin H. (1998). A novel class of evolutionarily conserved genes defined by piwi are essential for stem cell self-renewal. Genes Dev. 12, 3715–3727. 10.1101/gad.12.23.3715 9851978 PMC317255

[B35] Craig Venter J. Adams M. D. Myers E. W. Li P. W. Mural R. J. Sutton G. G. (2001). The sequence of the human genome. Science 291, 1304–1351. 10.1126/science.1058040 11181995

[B36] da Rocha S. T. Boeva V. Escamilla-Del-Arenal M. Ancelin K. Granier C. Matias N. R.. (2014). Jarid2 is implicated in the initial xist-induced targeting of PRC2 to the inactive X chromosome. Mol. Cell 53, 301–316. 10.1016/j.molcel.2014.01.002 24462204

[B37] Davidovich C. Zheng L. Goodrich K. J. Cech T. R. (2013). Promiscuous RNA binding by Polycomb repressive complex 2. Nat. Struct. Mol. Biol. 20, 1250–1257. 10.1038/nsmb.2679 24077223 PMC3823624

[B38] De Santa F. Barozzi I. Mietton F. Ghisletti S. Polletti S. Tusi B. K.. (2010). A large fraction of extragenic RNA pol II transcription sites overlap enhancers. PLoS Biol. 8:e1000384. 10.1371/journal.pbio.1000384 20485488 PMC2867938

[B39] Derrien T. Johnson R. Bussotti G. Tanzer A. Djebali S. Tilgner H.. (2012). The GENCODE v7 catalog of human long noncoding RNAs: analysis of their gene structure, evolution, and expression. Genome Res. 22, 1775–1789. 10.1101/gr.132159.111 22955988 PMC3431493

[B40] Di Ruscio A. Ebralidze A. K. Benoukraf T. Amabile G. Goff L. A. Terragni J.. (2013). DNMT1-interacting RNAs block gene-specific DNA methylation. Nature 503, 371–376. 10.1038/nature12598 24107992 PMC3870304

[B41] Dias B. G. Ressler K. J. (2014). Parental olfactory experience influences behavior and neural structure in subsequent generations. Nat. Neurosci. 17, 89–96. 10.1038/nn.3594 24292232 PMC3923835

[B42] Djebali S. Davis C. A. Merkel A. Dobin A. Lassmann T. Mortazavi A.. (2012). Landscape of transcription in human cells. Nature 489, 101–108. 10.1038/nature.11233 22955620 PMC3684276

[B43] Darwin C. (1859). On the Origin of Species by Means of Natural Selection, or, The preservation of Favoured Races in the Struggle for Life. London: John Murray. PMC5184128 30164232

[B44] ENCODE Project Consortium, Birney E. Stamatoyannopoulos J. A. Dutta A. Guig,ó R. Gingeras T. R.. (2007). Identification and analysis of functional elements in 1% of the human genome by the ENCODE pilot project. Nature 447, 799–816. 10.1038/nature05874 17571346 PMC2212820

[B45] Engreitz J. M. Haines J. E. Perez E. M. Munson G. Chen J. Kane M.. (2016a). Local regulation of gene expression by lncRNA promoters, transcription and splicing. Nature 539, 452–455. 10.1038/nature20149 27783602 PMC6853796

[B46] Engreitz J. M. Ollikainen N. Guttman M. (2016b). Long non-coding RNAs: spatial amplifiers that control nuclear structure and gene expression. Nat. Rev. Mol. Cell Biol. 17, 756–770. 10.1038/nrm.2016.126 27780979

[B47] Engreitz J. M. Pandya-Jones A. McDonel P. Shishkin A. Sirokman K. Surka C.. (2013). The Xist lncRNA exploits three-dimensional genome architecture to spread across the X chromosome. Science 341, 1237973–1237973. 10.1126/science.1237973 23828888 PMC3778663

[B48] Faghihi M. A. Modarresi F. Khalil A. M. Wood D. E. Sahagan B. G. Morgan T. E.. (2008). Expression of a noncoding RNA is elevated in Alzheimer's disease and drives rapid feed-forward regulation of β-secretase. Nat. Med. 14, 723–730. 10.1038/nm1784 18587408 PMC2826895

[B49] Fallmann J. Will S. Engelhardt J. Grüning B. Backofen R. Stadler P. F. (2017). Recent advances in RNA folding. J. Biotechnol. 261, 97–104. 10.1016/j.jbiotec.2017.07.007 28690134

[B50] Fatica A. Bozzoni I. (2014). Long non-coding RNAs: new players in cell differentiation and development. Nat. Rev. Genet. 15, 7–21. 10.1038/nrg3606 24296535

[B51] Feil R. Fraga M. F. (2012). Epigenetics and the environment: emerging patterns and implications. Nat. Rev. Genet. 13, 97–109. 10.1038/nrg3142 22215131

[B52] Felsenfeld G. (2014). A brief history of epigenetics. Cold Spring Harb. Perspect. Biol. 6:a018200. 10.1101/cshperspect.a018200 24384572 PMC3941222

[B53] Fire A. Xu S. Montgomery M. K. Kostas S. A. Driver S. E. Mello C. C. (1998). Potent and specific genetic interference by double-stranded RNA in Caenorhabditis elegans. Nature 391, 806–811. 10.1038/35888 9486653

[B54] Giorgetti L. Lajoie B. R. Carter A. C. Attia M. Zhan Y. Xu J.. (2016). Structural organization of the inactive X chromosome in the mouse. Nature 535, 575–579. 10.1038/nature18589 27437574 PMC5443622

[B55] Gloss B. S. Dinger M. E. (2016). The specificity of long noncoding RNA expression. Biochim. Biophys. Acta 1859, 16–22. 10.1016/j.bbagrm.2015.08.005 26297315

[B56] Gong C. Maquat L. E. (2011). lncRNAs transactivate STAU1-mediated mRNA decay by duplexing with 3′ UTRs via Alu elements. Nature 470, 284–288. 10.1038/nature09701 21307942 PMC3073508

[B57] Gong C. Li Z. Ramanujan K. Clay I. Zhang Y. Lemire-Brachat S.. (2015). A long non-coding RNA, LncMyoD, regulates skeletal muscle differentiation by blocking IMP2-mediated mRNA translation. Dev. Cell 34, 181–191. 10.1016/j.devcel.2015.05.009 26143994

[B58] Grossniklaus U. Kelly W. G. Kelly B. Ferguson-Smith A. C. Pembrey M. Lindquist S. (2013). Transgenerational epigenetic inheritance: how important is it? Nat. Rev. Genet. 14, 228–235. 10.1038/nrg3435 23416892 PMC4066847

[B59] Grote P. Wittler L. Hendrix D. Koch F. Währisch S. Beisaw A.. (2013). The tissue-specific lncRNA Fendrr is an essential regulator of heart and body wall development in the mouse. Dev. Cell 24, 206–214. 10.1016/j.devcel.2012.12.012 23369715 PMC4149175

[B60] Guil S. Soler M. Portela A. Carrère J. Fonalleras E. Gómez A.. (2012). Intronic RNAs mediate EZH2 regulation of epigenetic targets. Nat. Struct. Mol. Biol. 19, 664–670. 10.1038/nsmb.2315 22659877

[B61] Guttman M. Amit I. Garber M. French C. Lin M. F. Feldser D.. (2009). Chromatin signature reveals over a thousand highly conserved large non-coding RNAs in mammals. Nature 458, 223–227. 10.1038/nature07672 19182780 PMC2754849

[B62] Guttman M. Rinn J. L. (2012). Modular regulatory principles of large non-coding RNAs. Nature 482, 339–346. 10.1038/nature10887 22337053 PMC4197003

[B63] Guttman M. Russell P. Ingolia N. T. Weissman J. S. Lander E. S. (2013). Ribosome profiling provides evidence that large noncoding RNAs do not encode proteins. Cell 154, 240–251. 10.1016/j.cell.2013.06.009 23810193 PMC3756563

[B64] Hacisuleyman E. Goff L. A. Trapnell C. Williams A. Henao-Mejia J. Sun L.. (2014). Topological organization of multichromosomal regions by the long intergenic noncoding RNA Firre. Nat. Struct. Mol. Biol. 21, 198–206. 10.1038/nsmb.2764 24463464 PMC3950333

[B65] Haecker I. Renne R. (2014). HITS-CLIP and PAR-CLIP advance viral MiRNA targetome analysis. Crit. Rev. Eukaryot. Gene Expr. 24, 101–116. 10.1615/CritRevEukaryotGeneExpr.2014006367 24940765 PMC4062872

[B66] Hamilton A. J. Baulcombe D. C. (1999). A species of small antisense RNA in posttranscriptional gene silencing in plants. Science 286, 950–952. 10.1126/science.286.5441.950 10542148

[B67] Han P. Li W. Lin C. H. Yang J. Shang C. Nurnberg S. T.. (2014). A long noncoding RNA protects the heart from pathological hypertrophy. Nature 514, 102–106. 10.1038/nature13596 25119045 PMC4184960

[B68] Hansen T. B. Jensen T. I. Clausen B. H. Bramsen J. B. Finsen B. Damgaard C. K.. (2013). Natural RNA circles function as efficient microRNA sponges. Nature 495, 384–388. 10.1038/nature11993 23446346

[B69] Harrow J. Denoeud F. Frankish A. Reymond A. Chen C. K. Chrast J.. (2006). GENCODE: producing a reference annotation for ENCODE. Genome Biol. 7(Suppl. 1), S4.1–9. 10.1186/gb-2006-7-s1-s4 16925838 PMC1810553

[B70] Harrow J. Frankish A. Gonzalez J. M. Tapanari E. Diekhans M. Kokocinski F.. (2012). GENCODE: the reference human genome annotation for The ENCODE Project. Genome Res. 22, 1760–1774. 10.1101/gr.135350.111 22955987 PMC3431492

[B71] He D. C. Nickerson J. A. Penman S. (1990). Core filaments of the nuclear matrix. J. Cell Biol. 110, 569–580. 10.1083/jcb.110.3.569 2307700 PMC2116036

[B72] Heimans J. (1962). Hugo de vries and the gene concept. Am. Nat. 96, 93–104. 10.1086/282210

[B73] Hershey A. D. (1955). An upper limit to the protein content of the germinal substance of bacteriophage T2. Virology 1, 108–127. 10.1016/0042-6822(55)90009-X 13267980

[B74] Hoagland M. B. Stephenson M. L. Scott J. F. Hecht L. I. Zamecnik P. C. (1958). A soluble ribonucleic acid intermediate in protein synthesis. J. Biol. Chem. 231, 241–257. 13538965

[B75] Hogg J. R. Collins K. (2007). RNA-based affinity purification reveals 7SK RNPs with distinct composition and regulation. RNA 13, 868–880. 10.1261/rna.565207 17456562 PMC1869041

[B76] HSU T. C. (1962). Differential rate in RNA synthesis between euchromatin and heterochromatin. Exp. Cell Res. 27, 332–334. 10.1016/0014-4827(62)90238-0 14449550

[B77] Huarte M. Guttman M. Feldser D. Garber M. Koziol M. J. Kenzelmann-Broz D.. (2010). A large intergenic noncoding RNA induced by p53 mediates global gene repression in the p53 response. Cell 142, 409–419. 10.1016/j.cell.2010.06.040 20673990 PMC2956184

[B78] Huppertz I. Attig J. D'Ambrogio A. Easton L. E. Sibley C. R. Sugimoto Y.. (2014). iCLIP: protein-RNA interactions at nucleotide resolution. Methods 65, 274–287. 10.1016/j.ymeth.2013.10.011 24184352 PMC3988997

[B79] Jacob F. Monod J. (1961). Genetic regulatory mechanisms in the synthesis of proteins. J. Mol. Biol. 3, 318–356. 10.1016/S0022-2836(61)80072-7 13718526

[B80] Jacob F. Monod J. (1978). On the Regulation of Gene Activity, in Selected Papers in Molecular Biology, ed Monod J. (New York, NY: Academic Press), 472–490.

[B81] Jankowsky E. Harris M. E. (2015). Specificity and nonspecificity in RNA–protein interactions. Nat. Rev. Mol. Cell Biol. 16, 533–544. 10.1038/nrm4032 26285679 PMC4744649

[B82] Johannsen W. (1909). Elemente der Exakten Erblichkeitslehre. Deutsche Wesentlich Erweiterte Ausgabe in Fünfundzwanig Vorlesungen. Jena: G. Fischer

[B83] Johnsson P. Lipovich L. Grandér D. Morris K. V. (2014). Evolutionary conservation of long non-coding RNAs; sequence, structure, function. Biochim. Biophys. Acta 1840, 1063–1071. 10.1016/j.bbagen.2013.10.035 24184936 PMC3909678

[B84] Kalantry S. Magnuson T. (2006). The Polycomb group protein EED is dispensable for the initiation of random X-chromosome inactivation. PLoS Genet. 2:e66. 10.1371/journal.pgen.0020066 16680199 PMC1456320

[B85] Kaneko S. Son J. Shen S. S. Reinberg D. Bonasio R. (2013). PRC2 binds active promoters and contacts nascent RNAs in embryonic stem cells. Nat. Struct. Mol. Biol. 20, 1258–1264. 10.1038/nsmb.2700 24141703 PMC3839660

[B86] Kashi K. Henderson L. Bonetti A. Carninci P. (2016). Discovery and functional analysis of lncRNAs: methodologies to investigate an uncharacterized transcriptome. Biochim. Biophys. Acta 1859, 3–15. 10.1016/j.bbagrm.2015.10.010 26477492

[B87] Kawai J. Shinagawa A. Shibata K. Yoshino M. Itoh M. Ishii Y.. (2001). Functional annotation of a full-length mouse cDNA collection. Nature 409, 685–690. 10.1038/35055500 11217851

[B88] Khalil A. M. Guttman M. Huarte M. Garber M. Raj A. Rivea Morales D.. (2009). Many human large intergenic noncoding RNAs associate with chromatin-modifying complexes and affect gene expression. Proc. Natl. Acad. Sci. U.S.A. 106, 11667–11672. 10.1073/pnas.0904715106 19571010 PMC2704857

[B89] Kino T. Hurt D. E. Ichijo T. Nader N. Chrousos G. P. (2010). Noncoding RNA gas5 is a growth arrest- and starvation-associated repressor of the glucocorticoid receptor. Sci. Signal. 3:ra8. 10.1126/scisignal.2000568 20124551 PMC2819218

[B90] Klattenhoff C. A. Scheuermann J. C. Surface L. E. Bradley R. K. Fields P. A. Steinhauser M. L.. (2013). Braveheart, a long noncoding RNA required for cardiovascular lineage commitment. Cell 152, 570–583. 10.1016/j.cell.2013.01.003 23352431 PMC3563769

[B91] Korostowski L. Sedlak N. Engel N. (2012). The Kcnq1ot1 long non-coding RNA affects chromatin conformation and expression of Kcnq1, but does not regulate its imprinting in the developing heart. PLoS Genet. 8:e1002956 10.1371/journal.pgen.1002956 23028363 PMC3447949

[B92] König J. Zarnack K. Rot G. Curk T. Kayikci M. Zupan B.. (2010). iCLIP reveals the function of hnRNP particles in splicing at individual nucleotide resolution. Nat. Struct. Mol. Biol. 17, 909–915. 10.1038/nsmb.1838 20601959 PMC3000544

[B93] Kretz M. Siprashvili Z. Chu C. Webster D. E. Zehnder A. Qu K.. (2013). Control of somatic tissue differentiation by the long non-coding RNA TINCR. Nature 493, 231–235. 10.1038/nature11661 23201690 PMC3674581

[B94] Kruger K. Grabowski P. J. Zaug A. J. Sands J. Gottschling D. E. Cech T. R. (1982). Self-splicing RNA: autoexcision and autocyclization of the ribosomal RNA intervening sequence of Tetrahymena. Cell 31, 147–157. 10.1016/0092-8674(82)90414-7 6297745

[B95] Kung J. T. Colognori D. Lee J. T. (2013). Long noncoding RNAs: past, present, and future. Genetics 193, 651–669. 10.1534/genetics.112.146704 23463798 PMC3583990

[B96] Lander E. S. Linton L. M. Birren B. Nusbaum C. Zody M. C. Baldwin J.. (2001). Initial sequencing and analysis of the human genome. Nature 409, 860–921. 10.1038/35057062 11237011

[B97] Lanz R. B. McKenna N. J. Onate S. A. Albrecht U. Wong J. Tsai S. Y.. (1999). A steroid receptor coactivator, SRA, functions as an RNA and is present in an SRC-1 complex. Cell 97, 17–27. 10.1016/S0092-8674(00)80711-4 10199399

[B98] Leamy K. A. Yennawar N. H. Bevilacqua P. C. (2017). Cooperative RNA folding under cellular conditions arises from both tertiary structure stabilization and secondary structure destabilization. Biochemistry 56, 3422–3433. 10.1021/acs.biochem.7b00325 28657303 PMC5542450

[B99] Lee H. Y. Haurwitz R. E. Apffel A. Zhou K. Smart B. Wenger C. D.. (2013). RNA-protein analysis using a conditional CRISPR nuclease. Proc. Natl. Acad. Sci. U.S.A. 110, 5416–5421. 10.1073/pnas.1302807110 23493562 PMC3619310

[B100] Lee R. C. Feinbaum R. L. Ambros V. (1993). The, C. elegans heterochronic gene lin-4 encodes small RNAs with antisense complementarity to lin-14. Cell 75, 843–854. 10.1016/0092-8674(93)90529-Y 8252621

[B101] Legnini I. Morlando M. Mangiavacchi A. Fatica A. Bozzoni I. (2014). A feedforward regulatory loop between HuR and the long noncoding RNA linc-MD1 controls early phases of myogenesis. Mol. Cell 53, 506–514. 10.1016/j.molcel.2013.12.012 24440503 PMC3919156

[B102] Leppek K. Stoecklin G. (2014). An optimized streptavidin-binding RNA aptamer for purification of ribonucleoprotein complexes identifies novel ARE-binding proteins. Nucleic Acids Res. 42, e13–e13. 10.1093/nar/gkt956 24157833 PMC3902943

[B103] Licatalosi D. D. Mele A. Fak J. J. Ule J. Kayikci M. Chi S. W.. (2008). HITS-CLIP yields genome-wide insights into brain alternative RNA processing. Nature 456, 464–469. 10.1038/nature07488 18978773 PMC2597294

[B104] Lindblad-Toh K. Wade C. M. Mikkelsen T. S. Karlsson E. K. Jaffe D. B. Kamal M.. (2005). Genome sequence, comparative analysis and haplotype structure of the domestic dog. Nature 438, 803–819. 10.1038/nature04338 16341006

[B105] Lingner J. Cech T. R. (1996). Purification of telomerase from Euplotes aediculatus: requirement of a primer 3′ overhang. Proc. Natl. Acad. Sci. U.S.A. 93, 10712–10717. 10.1073/pnas.93.20.10712 8855245 PMC38220

[B106] Liu Y. W. Xia R. Lu K. Xie M. Yang F. Sun M.. (2017). LincRNAFEZF1-AS1 represses p21 expression to promote gastric cancer proliferation through LSD1-Mediated H3K4me2 demethylation. Mol. Cancer 16:39. 10.1186/s12943-017-0588-9 28209170 PMC5314465

[B107] McHugh C. A. Chen C. K. Chow A. Surka C. F. Tran C. McDonel P.. (2015). The Xist lncRNA interacts directly with SHARP to silence transcription through HDAC3. Nature 521, 232–236. 10.1038/nature14443 25915022 PMC4516396

[B108] Meller J. Porollo A. (2012). Chapter 1 Computational methods for prediction of protein-protein interaction sites, in Protein-Protein Interactions - Computational and Experimental Tools, ed Cai W. (InTech).

[B109] Mendel G. (1866). Versuche über Pflanzen-Hybriden. Brünn: Im Verlage des Vereines.

[B110] Memczak S. Jens M. Elefsinioti A. Torti F. Krueger J. Rybak A.. (2013). Circular RNAs are a large class of animal RNAs with regulatory potency. Nature 495, 333–338. 10.1038/nature11928 23446348

[B111] Mercer T. R. Mattick J. S. (2013). Structure and function of long noncoding RNAs in epigenetic regulation. Nat. Struct. Mol. Biol. 20, 300–307. 10.1038/nsmb.2480 23463315

[B112] Mercer T. R. Gerhardt D. J. Dinger M. E. Crawford J. Trapnell C. Jeddeloh J. A.. (2011). Targeted RNA sequencing reveals the deep complexity of the human transcriptome. Nat. Biotechnol. 30, 99–104. 10.1038/nbt.2024 22081020 PMC3710462

[B113] Mili S. (2004). Evidence for reassociation of RNA-binding proteins after cell lysis: implications for the interpretation of immunoprecipitation analyses. RNA 10, 1692–1694. 10.1261/rna.7151404 15388877 PMC1370654

[B114] Miska E. A. Ferguson-Smith A. C. (2016). Transgenerational inheritance: models and mechanisms of non-DNA sequence-based inheritance. Science 354, 59–63. 10.1126/science.aaf4945 27846492

[B115] Morgan T. H. Sturtevant A. H. Muller H. J. Bridges C. B. (1915). Mechanism of mendelian heridity. Trans. Am. Microsc. Soc. 34:293 10.2307/3221480

[B116] Morlando M. Ballarino M. Fatica A. Bozzoni I. (2014). The Role of long noncoding RNAs in the epigenetic control of gene expression. Chem. Med. Chem. 9, 505–510. 10.1002/cmdc.201300569 24488863

[B117] Morozova O. Marra M. A. (2008). Applications of next-generation sequencing technologies in functional genomics. Genomics 92, 255–264. 10.1016/j.ygeno.2008.07.001 18703132

[B118] Moseley M. L. Zu T. Ikeda Y. Gao W. Mosemiller A. K. Daughters R. S.. (2006). Bidirectional expression of CUG and CAG expansion transcripts and intranuclear polyglutamine inclusions in spinocerebellar ataxia type 8. Nat. Genet. 38, 758–769. 10.1038/ng1827 16804541

[B119] Mousavi K. Zare H. Dell'orso S. Grontved L. Gutierrez-Cruz G. Derfoul A.. (2013). eRNAs promote transcription by establishing chromatin accessibility at defined genomic loci. Mol. Cell 51, 606–617. 10.1016/j.molcel.2013.07.022 23993744 PMC3786356

[B120] Mousavi K. Zare H. Koulnis M. Sartorelli V. (2014). The emerging roles of eRNAs in transcriptional regulatory networks. RNA Biol. 11, 106–110. 10.4161/rna.27950 24525859 PMC3973729

[B121] Mouse Genome Sequencing Consortium, Waterston R. H. Lindblad-Toh K. Birney E. Rogers J. Abril J. F.. (2002). Initial sequencing and comparative analysis of the mouse genome. Nature 420, 520–562. 10.1038/nature01262 12466850

[B122] Mudge J. M. Harrow J. (2015). Creating reference gene annotation for the mouse C57BL6/J genome assembly. Mamm. Genome 26, 366–378. 10.1007/s00335-015-9583-x 26187010 PMC4602055

[B123] Mueller A. C. Cichewicz M. A. Dey B. K. Layer R. Reon B. J. Gagan J. R.. (2015). MUNC, a long noncoding RNA that facilitates the function of MyoD in skeletal myogenesis. Mol. Cell. Biol. 35, 498–513. 10.1128/MCB.01079-14 25403490 PMC4285423

[B124] Muller H. J. (1930). Types of visible variations induced by X-rays inDrosophila. J. Genet. 22, 299–334. 10.1007/BF02984195

[B125] Muppirala U. K. Lewis B. A. Dobbs D. (2013). Computational tools for investigating RNA-protein interaction partners. J. Comput. Sci. Syst. Biol. 6, 182–187. 10.4172/jcsb.1000115

[B126] Nakagawa S. Naganuma T. Shioi G. Hirose T. (2011). Paraspeckles are subpopulation-specific nuclear bodies that are not essential in mice. J. Cell Biol. 193, 31–39. 10.1083/jcb.201011110 21444682 PMC3082198

[B127] Nelson B. R. Makarewich C. A. Anderson D. M. Winders B. R. Troupes C. D. Wu F.. (2016). A peptide encoded by a transcript annotated as long noncoding RNA enhances SERCA activity in muscle. Science 351, 271–275. 10.1126/science.aad4076 26816378 PMC4892890

[B128] Nickerson J. A. Krochmalnic G. Wan K. M. Penman S. (1989). Chromatin architecture and nuclear RNA. Proc. Natl. Acad. Sci. U.S.A. 86, 177–181. 10.1073/pnas.86.1.177 2911567 PMC286427

[B129] Nitsche A. Stadler P. F. (2017). Evolutionary clues in lncRNAs. Wiley Interdiscip. Rev. RNA 8:e1376. 10.1002/wrna.1376 27436689

[B130] Niu D. K. Jiang L. (2013). Can ENCODE tell us how much junk DNA we carry in our genome? Biochem. Biophys. Res. Commun. 430, 1340–1343. 10.1016/j.bbrc.2012.12.074 23268340

[B131] Ohno S. (1972). So much “junk” DNA in our genome. Brookhaven Symp. Biol. 23, 366–370. 5065367

[B132] Ounzain S. Pedrazzini T. (2015). The promise of enhancer-associated long noncoding RNAs in cardiac regeneration. Trends Cardiovasc. Med. 25, 592–602. 10.1016/j.tcm.2015.01.014 25753179

[B133] Ounzain S. Micheletti R. Arnan C. Plaisance I. Cecchi D. Schroen B.. (2015). CARMEN, a human super enhancer-associated long noncoding RNA controlling cardiac specification, differentiation and homeostasis. J. Mol. Cell. Cardiol. 89, 98–112. 10.1016/j.yjmcc.2015.09.016 26423156

[B134] Pandey R. R. Mondal T. Mohammad F. Enroth S. Redrup L. Komorowski J.. (2008). Kcnq1ot1 antisense noncoding rna mediates lineage-specific transcriptional silencing through chromatin-level regulation. Mol. Cell 32, 232–246. 10.1016/j.molcel.2008.08.022 18951091

[B135] Peritz T. Zeng F. Kannanayakal T. J. Kilk K. Eiríksdóttir E. Langel U.. (2006). Immunoprecipitation of mRNA-protein complexes. Nat. Protoc. 1, 577–580. 10.1038/nprot.2006.82 17406284

[B136] Pintacuda G. Young A. N. Cerase A. (2017). Function by structure: spotlights on xist long non-coding RNA. Front. Mol. Biosci. 4:90. 10.3389/fmolb.2017.00090 29302591 PMC5742192

[B137] Piwecka M. GlaŽar P. Hernandez-Miranda L. R. Memczak S. Wolf S. A. Rybak-Wolf A.. (2017). Loss of a mammalian circular RNA locus causes miRNA deregulation and affects brain function. Science 357:eaam8526. 10.1126/science.aam8526 28798046

[B138] Plath K. Fang J. Mlynarczyk-Evans S. K. Cao R. Worringer K. A. Wang H.. (2003). Role of histone H3 lysine 27 methylation in X inactivation. Science 300, 131–135. 10.1126/science.1084274 12649488

[B139] Portoso M. Ragazzini R. Brencic Ž. Moiani A. Michaud A. Vassilev I.. (2017). PRC2 is dispensable for HOTAIR-mediated transcriptional repression. EMBO J. 36, 981–994. 10.15252/embj.201695335 28167697 PMC5391141

[B140] Ribeiro D. M. Zanzoni A. Cipriano A. Delli Ponti R. Spinelli L. Ballarino M.. (2017). Protein complex scaffolding predicted as a prevalent function of long non-coding RNAs. Nucleic Acids Res. 46, 917–928. 10.1093/nar/gkx1169 29165713 PMC5778612

[B141] Rinn J. L. Chang H. Y. (2012). Genome regulation by long noncoding RNAs. Annu. Rev. Biochem. 81, 145–166. 10.1146/annurev-biochem-051410-092902 22663078 PMC3858397

[B142] Rinn J. L. Kertesz M. Wang J. K. Squazzo S. L. Xu X. Brugmann S. A.. (2007). Functional demarcation of active and silent chromatin domains in human HOX loci by noncoding RNAs. Cell 129, 1311–1323. 10.1016/j.cell.2007.05.022 17604720 PMC2084369

[B143] Ripoche M. A. Kress C. Poirier F. Dandolo L. (1997). Deletion of the H19 transcription unit reveals the existence of a putative imprinting control element. Genes Dev. 11, 1596–1604. 10.1101/gad.11.12.1596 9203585

[B144] Sauvageau M. Goff L. A. Lodato S. Bonev B. Groff A. F. Gerhardinger C.. (2013). Multiple knockout mouse models reveal lincRNAs are required for life and brain development. Elife 2:e01749. 10.7554/eLife.01749 24381249 PMC3874104

[B145] Scherrer K. Darnell J. E. (1962). Sedimentation characteristics of rapidly labelled RNA from HeLa cells. Biochem. Biophys. Res. Commun. 7, 486–490. 10.1016/0006-291X(62)90341-8 14498283

[B146] Scherrer K. Latham H. Darnell J. E. (1963). Demonstration of an unstable RNA and of a precursor to ribosomal RNA in HeLa cells. Proc. Natl. Acad. Sci. U.S.A. 49, 240–248. 10.1073/pnas.49.2.240 13991616 PMC299789

[B147] Schoeftner S. Sengupta A. K. Kubicek S. Mechtler K. Spahn L. Koseki H.. (2006). Recruitment of PRC1 function at the initiation of X inactivation independent of PRC2 and silencing. EMBO J. 25, 3110–3122. 10.1038/sj.emboj.7601187 16763550 PMC1500994

[B148] Shi X. Sun M. Liu H. Yao Y. Song Y. (2013). Long non-coding RNAs: a new frontier in the study of human diseases. Cancer Lett. 339, 159–166. 10.1016/j.canlet.2013.06.013 23791884

[B149] Shiraki T. Kondo S. Katayama S. Waki K. Kasukawa T. Kawaji H.. (2003). Cap analysis gene expression for high-throughput analysis of transcriptional starting point and identification of promoter usage. Proc. Natl. Acad. Sci. U.S.A. 100, 15776–15781. 10.1073/pnas.2136655100 14663149 PMC307644

[B150] Slobodin B. Gerst J. E. (2010). A novel mRNA affinity purification technique for the identification of interacting proteins and transcripts in ribonucleoprotein complexes. RNA 16, 2277–2290. 10.1261/rna.2091710 20876833 PMC2957065

[B150a] Schroeder R. Grossberger R. Pichler A. Waldsich C. (2002). RNA folding *in vivo*. Curr. Opin. Struct. Biol. 12, 296–300. 12127447 10.1016/s0959-440x(02)00325-1

[B151] Spitzer J. Hafner M. Landthaler M. Ascano M. Farazi T. Wardle G.. (2014). PAR-CLIP (Photoactivatable Ribonucleoside-Enhanced Crosslinking and Immunoprecipitation): a step-by-step protocol to the transcriptome-wide identification of binding sites of RNA-binding proteins. Meth. Enzymol. 539, 113–161. 10.1016/B978-0-12-420120-0.00008-6 24581442 PMC4180672

[B152] Splinter E. de Wit E. Nora E. P. Klous P. van de Werken H. J. G. Zhu Y. (2011). The inactive X chromosome adopts a unique three-dimensional conformation that is dependent on Xist, R. N. A. Genes Dev. 25, 1371–1383. 10.1101/gad.633311 21690198 PMC3134081

[B153] Srisawat C. Engelke D. R. (2001). Streptavidin aptamers: affinity tags for the study of RNAs and ribonucleoproteins. RNA 7, 632–641. 10.1017/S135583820100245X 11345441 PMC1370116

[B154] Struhl K. (2007). Transcriptional noise and the fidelity of initiation by RNA polymerase II. Nat. Struct. Mol. Biol. 14, 103–105. 10.1038/nsmb0207-103 17277804

[B155] Suresh V. Liu L. Adjeroh D. Zhou X. (2015). RPI-Pred: predicting ncRNA-protein interaction using sequence and structural information. Nucleic Acids Res. 43, 1370–1379. 10.1093/nar/gkv020 25609700 PMC4330382

[B156] Szyf M. (2013). Lamarck revisited: epigenetic inheritance of ancestral odor fear conditioning. Nat. Neurosci. 17, 2–4. 10.1038/nn.3603 24369368

[B157] Tang J. Y. Lee J. C. Chang Y. T. Hou M. F. Huang H. W. Liaw C. C.. (2013). Long noncoding RNAs-related diseases, cancers, and drugs. Sci.World J. 2013:943539. 10.1155/2013/943539 23843741 PMC3690748

[B158] Tsai B. P. Wang X. Huang L. Waterman M. L. (2011). Quantitative profiling of *in vivo*-assembled RNA-protein complexes using a novel integrated proteomic approach. Mol. Cell Proteomics 10:M110.007385. 10.1074/mcp.M110.007385 21285413 PMC3069349

[B159] Tsai M.-C. Manor O. Wan Y. Mosammaparast N. Wang J. K. Lan F.. (2010). Long noncoding RNA as modular scaffold of histone modification complexes. Science 329, 689–693. 10.1126/science.1192002 20616235 PMC2967777

[B160] Uchida S. Dimmeler S. (2015). Long noncoding RNAs in cardiovascular diseases. Circ. Res. 116, 737–750. 10.1161/CIRCRESAHA.116.302521 25677520

[B161] Ule J. Jensen K. B. Ruggiu M. Mele A. Ule A. Darnell R. B. (2003). CLIP identifies Nova-regulated RNA networks in the brain. Science 302, 1212–1215. 10.1126/science.1090095 14615540

[B162] Van Nostrand E. L. Pratt G. A. Shishkin A. A. Gelboin-Burkhart C. Fang M. Y. Sundararaman B.. (2016). Robust transcriptome-wide discovery of RNA-binding protein binding sites with enhanced CLIP (eCLIP). Nat. Methods 13, 508–514. 10.1038/nmeth.3810 27018577 PMC4887338

[B163] Waddington C. H. (1940). (1) Principles of Development (2) Les progrès récents de l'embryologie expérimentale. Nature 145, 952–953. 10.1038/145952a0

[B164] Wade M. J. Dawkins R. (1978). The selfish gene. Evolution 32:220.

[B165] Wang J. Gong C. Maquat L. E. (2013). Control of myogenesis by rodent SINE-containing lncRNAs. Genes Dev. 27, 793–804. 10.1101/gad.212639.112 23558772 PMC3639419

[B166] Wang K. C. Yang Y. W. Liu B. Sanyal A. Corces-Zimmerman R. Chen Y.. (2011). A long noncoding RNA maintains active chromatin to coordinate homeotic gene expression. Nature 472, 120–124. 10.1038/nature09819 21423168 PMC3670758

[B167] Wang X. Arai S. Song X. Reichart D. Du K. Pascual G.. (2008). Induced ncRNAs allosterically modify RNA-binding proteins in cis to inhibit transcription. Nature 454, 126–130. 10.1038/nature06992 18509338 PMC2823488

[B168] Wang Z. Tollervey J. Briese M. Turner D. Ule J. (2009). CLIP: construction of cDNA libraries for high-throughput sequencing from RNAs cross-linked to proteins *in vivo*. Methods 48, 287–293. 10.1016/j.ymeth.2009.02.021 19272451

[B169] Wang Z. Zhang X. J. Ji Y. X. Zhang P. Deng K. Q. Gong J.. (2016). The long noncoding RNA Chaer defines an epigenetic checkpoint in cardiac hypertrophy. Nat. Med. 22, 1131–1139. 10.1038/nm.4179 27618650 PMC5053883

[B170] Wassarman D. A. Steitz J. A. (1992). Interactions of small nuclear RNA's with precursor messenger RNA during *in vitro* splicing. Science 257, 1918–1925. 10.1126/science.1411506 1411506

[B171] Watson J. D. Crick F. H. C. (1953). Molecular structure of nucleic acids: a structure for deoxyribose nucleic acid. Nature 171, 737–738. 10.1038/171737a0 13054692

[B172] Weaver I. C. Cervoni N. Champagne F. A. D'Alessio A. C. Sharma S. Seckl J. R.. (2004). Epigenetic programming by maternal behavior. Nat. Neurosci. 7, 847–854. 10.1038/nn1276 15220929

[B173] Wutz A. (2011). Gene silencing in X-chromosome inactivation: advances in understanding facultative heterochromatin formation. Nat. Rev. Genet. 12, 542–553. 10.1038/nrg3035 21765457

[B174] Wutz A. Rasmussen T. P. Jaenisch R. (2002). Chromosomal silencing and localization are mediated by different domains of Xist, R. N. A. Nat. Genet. 30, 167–174. 10.1038/ng820 11780141

[B175] Xue Z. Hennelly S. Doyle B. Gulati A. A. Novikova I. V. Sanbonmatsu K. Y.. (2016). A G-Rich Motif in the lncRNA braveheart interacts with a zinc-finger transcription factor to specify the cardiovascular lineage. Mol. Cell 64, 37–50. 10.1016/j.molcel.2016.08.010 27618485 PMC6728430

[B176] Yang J. H. Li J. H. Shao P. Zhou H. Chen Y. Q. Qu L. H. (2011). starBase: a database for exploring microRNA-mRNA interaction maps from Argonaute CLIP-Seq and Degradome-Seq data. Nucleic Acids Res. 39, D202–D209. 10.1093/nar/gkq1056 21037263 PMC3013664

[B177] Yang Y. W. Flynn R. A. Chen Y. Qu K. Wan B. Wang K. C.. (2014). Essential role of lncRNA binding for WDR5 maintenance of active chromatin and embryonic stem cell pluripotency. Elife 3:183. 10.7554/eLife.02046 24521543 PMC3921674

[B178] Yang Y. C. T. Di C. Hu B. Zhou M. Liu Y. Song N.. (2015). CLIPdb: a CLIP-seq database for protein-RNA interactions. BMC Genomics 16:51. 10.1186/s12864-015-1273-2 25652745 PMC4326514

[B179] Yoon J. H. Abdelmohsen K. Srikantan S. Yang X. Martindale J. L. De S.. (2012). LincRNA-p21 suppresses target mRNA translation. Mol. Cell 47, 648–655. 10.1016/j.molcel.2012.06.027 22841487 PMC3509343

[B180] Yuan G. C. (2012). Linking genome to epigenome. Wiley Interdiscip. Rev. Syst. Biol. Med. 4, 297–309. 10.1002/wsbm.1165 22344857 PMC3328655

[B181] Zeng F. Peritz T. Kannanayakal T. J. Kilk K. Eiríksdóttir E. Langel U.. (2006). A protocol for PAIR: PNA-assisted identification of RNA binding proteins in living cells. Nat. Protoc. 1, 920–927. 10.1038/nprot.2006.81 17406325

[B182] Zhang B. Arun G. Mao Y. S. Lazar Z. Hung G. Bhattacharjee G.. (2012). The lncRNA Malat1 is dispensable for mouse development but its transcription plays a cis-regulatory role in the adult. Cell Rep. 2, 111–123. 10.1016/j.celrep.2012.06.003 22840402 PMC3408587

[B183] Zhang H. Zeitz M. J. Wang H. Niu B. Ge S. Li W.. (2014). Long noncoding RNA-mediated intrachromosomal interactions promote imprinting at the Kcnq1 locus. J. Cell Biol. 204, 61–75. 10.1083/jcb.201304152 24395636 PMC3882787

[B184] Zhang X. Wu D. Chen L. Li X. Yang J. Fan D.. (2014). RAID: a comprehensive resource for human RNA-associated (RNA-RNA/RNA-protein) interaction. RNA 20, 989–993. 10.1261/rna.044776.114 24803509 PMC4114696

[B185] Zhao J. Sun B. K. Erwin J. A. Song J. J. Lee J. T. (2008). Polycomb proteins targeted by a short repeat RNA to the mouse X chromosome. Science 322, 750–756. 10.1126/science.1163045 18974356 PMC2748911

[B186] Zielinski J. Kilk K. Peritz T. Kannanayakal T. Miyashiro K. Y. Eiríksdóttir E.. (2006). *In vivo* identification of ribonucleoprotein-RNA interactions. Proc. Natl. Acad. Sci. U.S.A. 103, 1557–1562. 10.1073/pnas.0510611103 16432185 PMC1345716

